# Data on changes in lipid profiles during the differentiation and maturation of human subcutaneous white adipocytes analyzed using chromatographic and bioinformatic tools

**DOI:** 10.1016/j.dib.2022.108245

**Published:** 2022-05-06

**Authors:** Aya Kitamoto, Takuya Kitamoto

**Affiliations:** Advanced Research Facilities and Services, Hamamatsu University School of Medicine, Handayama, Higashi-ku, Hamamatsu 431-3192, Japan

**Keywords:** Human subcutaneous white preadipocytes, Lipid profile, Liquid chromatography-mass spectrometry, LipidSearch, MetaboAnalyst

## Abstract

In this dataset, we have described the changes in the lipid profile occurring during the differentiation and maturation of cultured human subcutaneous white preadipocytes into mature adipocytes. We divided three cell lines of Caucasian-derived subcutaneous preadipocytes into five stages (stage-1 to stage-5), from subcutaneous preadipocytes to mature subcutaneous adipocytes filled with many lipid droplets. Lipids were extracted from the cells at each stage by employing the Bligh and Dyer method and processed using untargeted liquid chromatography coupled with Q-Exactive Orbitrap tandem mass spectrometry. The lipids were identified using LipidSearch 4.2.13, and statistical analysis was performed using MetaboAnalyst 5.0. Dendrogram and principal component analysis clearly separated different stages of cells such as subcutaneous preadipocytes (stage-1), after the induction of differentiation into adipocytes (stage-2), and after the start of fat accumulation (stage-3 to stage-5). Of the 309 lipid species detected in LipidSearch 4.2.13, a total of 145 were statistically significant (false discovery rate < 0.05). The data are available at Metabolomics Workbench, Study ID ST001958: [https://www.metabolomicsworkbench.org/data/DRCCMetadata.php?Mode=Project&ProjectID=PR001245].

## Specifications Table

**Table:** 

Subject	Omics: Lipidomics
Specific subject area	Changes in lipid profile during subcutaneous adipocyte differentiation and maturation
Type of data	Chromatograms Tables Figures
How the data were acquired	Using liquid Chromatography Q-Exactive Orbitrap Mass Spectrometry system, Xcalibur 4.3.73.11, LipidSearch 4.2.13, and MetaboAnalyst 5.0.
Data format	Raw Analyzed Filtered
Parameters for data collection	Subcutaneous preadipocytes (stage-1), after inducing differentiation into adipocytes (stage-2), after the initiation of fat accumulation, the early stage (stage-3), the middle stage (stage-4), and mature subcutaneous adipocytes (stage-5). Stages-2, -3, -4, and -5 are days 3, 6, 8, and 10 after the induction of differentiation, respectively.
Description of data collection	Three cell lines of Caucasian-derived subcutaneous preadipocytes were divided into five stages of differentiation (stage-1 to stage-5) for 15 samples. After chloroform–methanol-based extraction, lipid samples were analyzed using chromatographic analysis coupled with mass spectroscopy. Data were acquired using Xcalibur 4.3.73.11 and analyzed using LipidSearch 4.2.13 and MetaboAnalyst 5.0.
Data source location	Advanced Research Facilities and Services, Hamamatsu University School of Medicine, Hamamatsu/Higashi-ku/Handayama, Japan
Data accessibility	Repository name: Metabolomics Workbench- Project ID PR001245 Data identification number: http://dx.doi.org/10.21228/M8S985 Direct URL to data: https://www.metabolomicsworkbench.org/data/DRCCMetadata.php?Mode=Project&ProjectID=PR001245

## Value of the Data

• This dataset is useful for detailed understanding of the changes in the lipid profiles occurring during the differentiation and maturation of three cell lines of Caucasian-derived subcutaneous preadipocytes (stage-1) divided into five stages: differentiation into adipocytes (stage-2) and post-differentiation (stage-3 to -5) to become mature adipocytes filled with many lipid droplets in the cytoplasm.
• These data would benefit researchers interested in obesity and obesity-related metabolic diseases to explore the clinical, diagnostic, and treatment targets.
• These data can be applied to the development of a database for future lipidomics experiments of obesity and obesity-related metabolic diseases and can be integrated with other omics methods to improve our understanding of changes in the lipid profiles occurring in adipose tissues.

## 1 Data Description

**Fig. 1:**
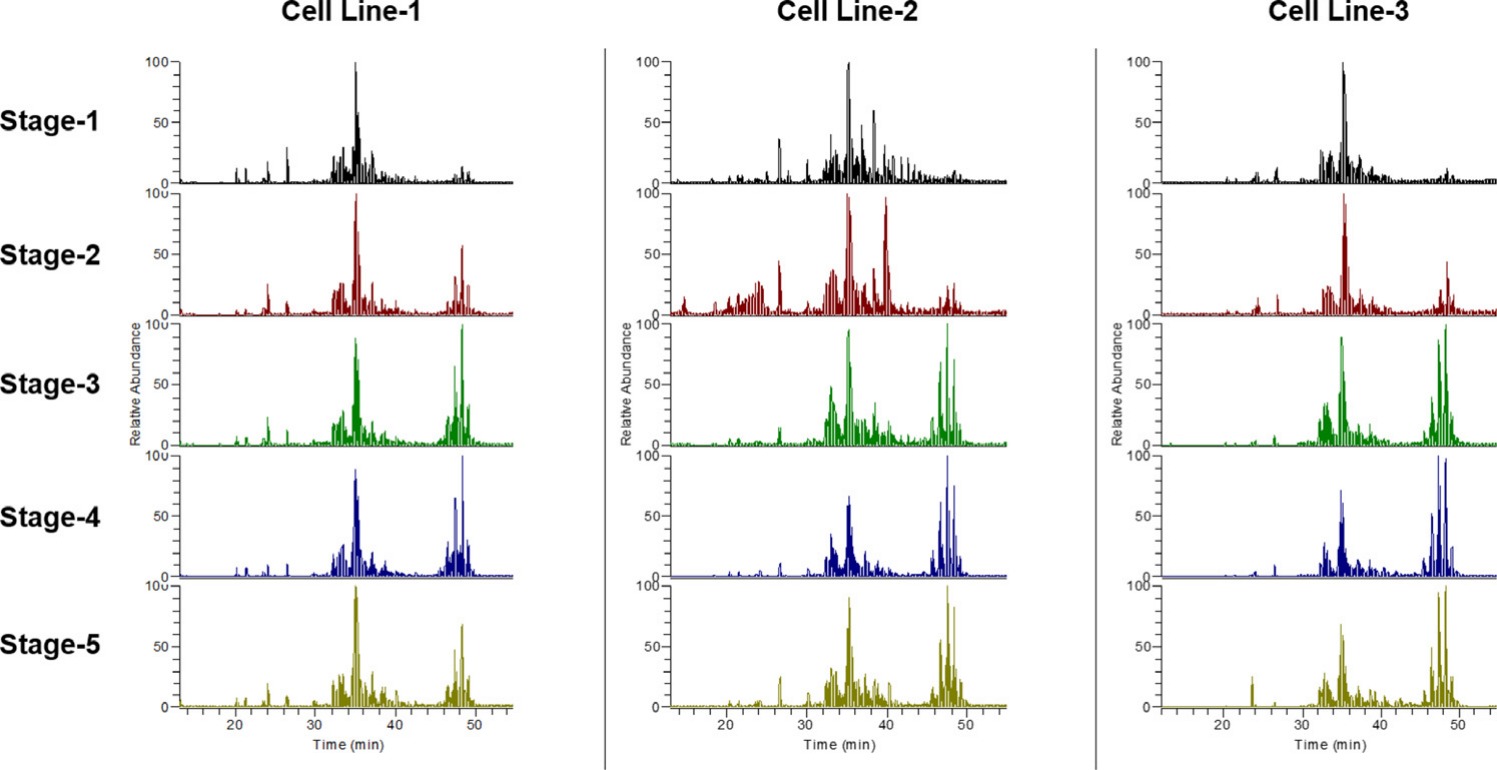
Total ion chromatogram of three lines of Caucasian-derived subcutaneous preadipocytes, each with five stages. Data collection was performed using Xcalibur 4.3.73.11.

**Fig. 2:**
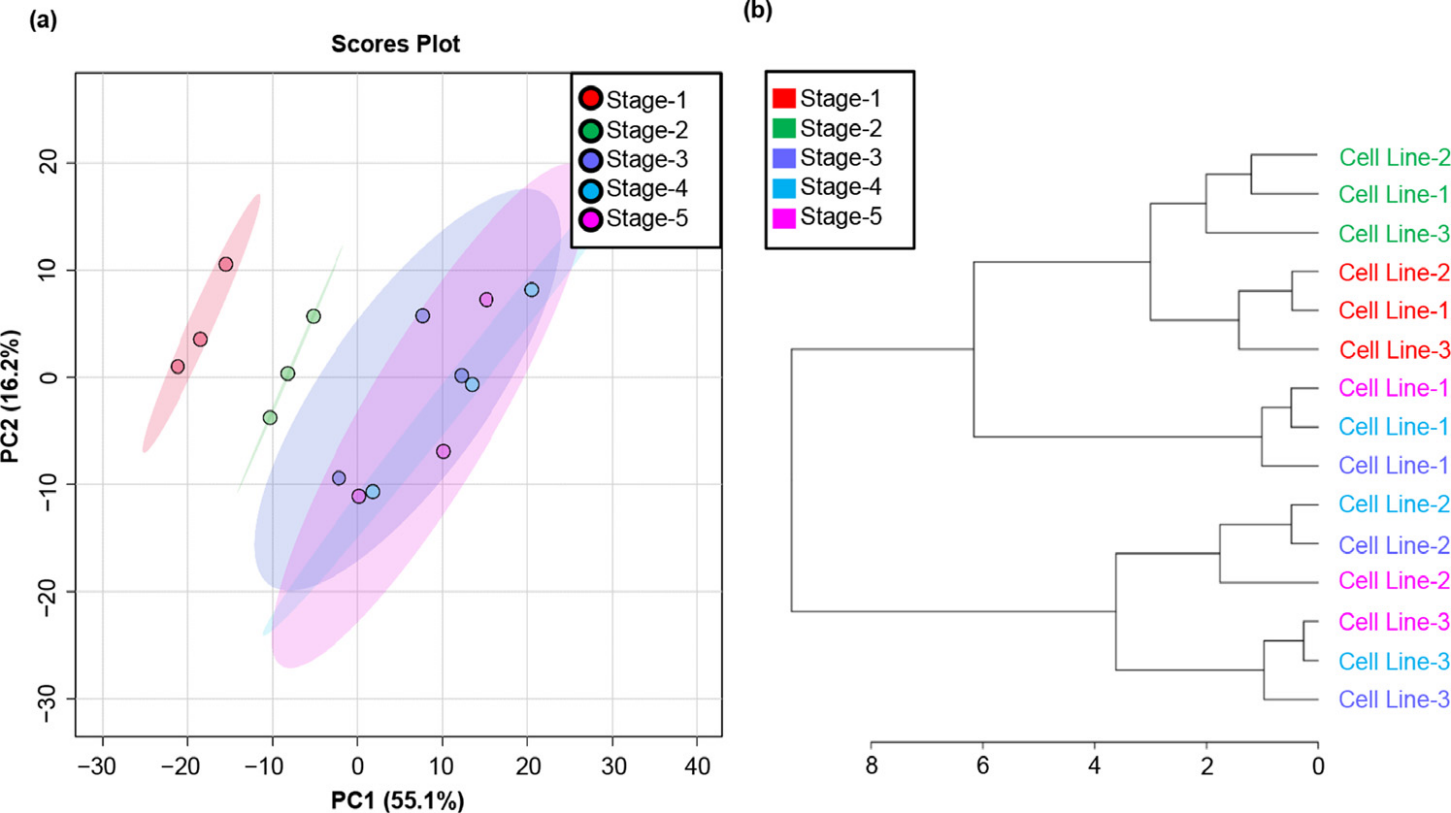
(a) Principal component analysis of samples plotted against their projections onto PC1 and PC2. (b) Dendrogram showing the relationship between lipid species at five stages using Pearson distances and ward clustering. Both figures were generated using MetaboAnalyst 5.0.

**Fig. 3:**
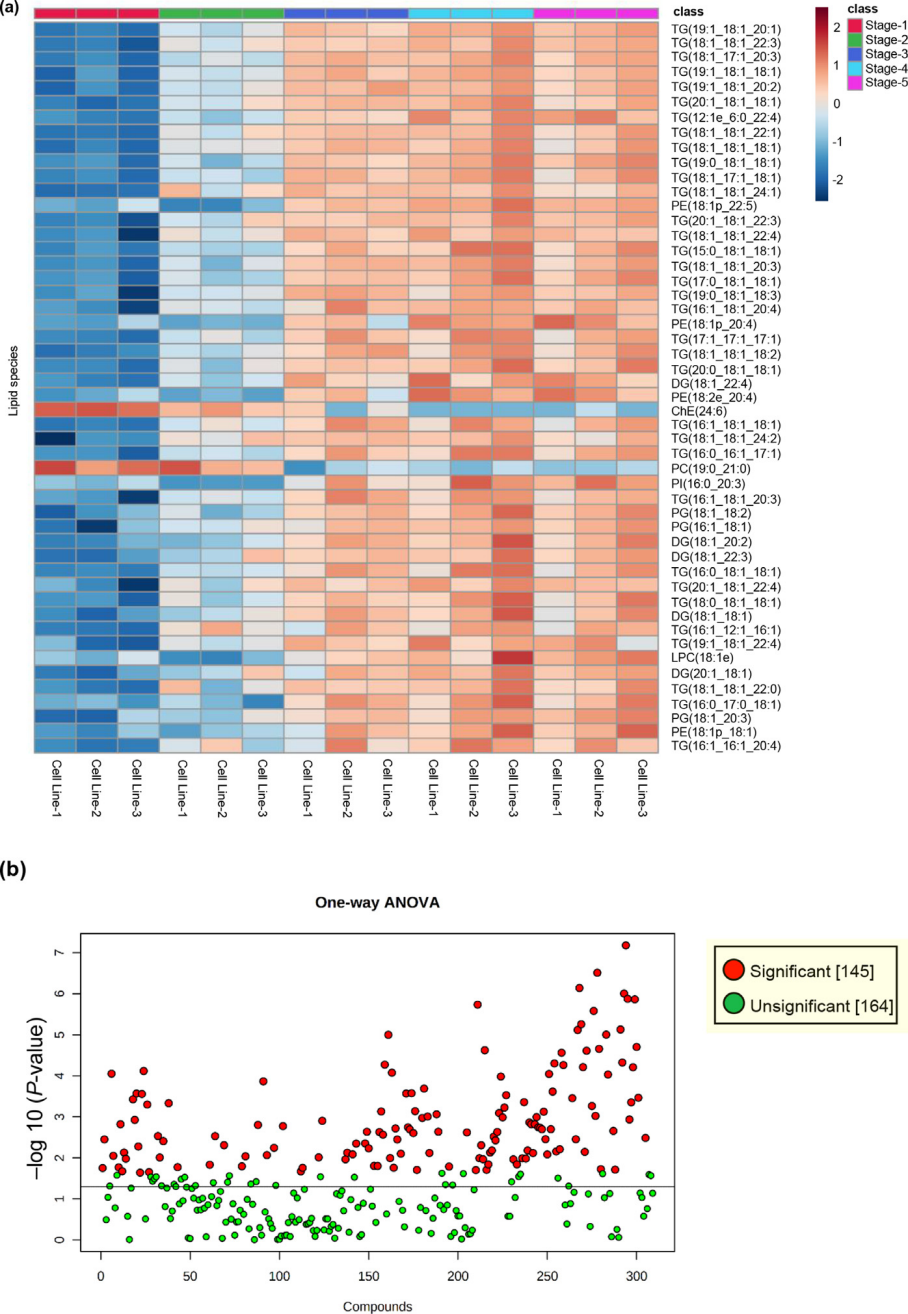
(a) Hierarchical clustering heatmap analysis among the stages was performed using Ward's method with the Euclidean distance. Heatmaps showing the top 50 lipid species that differed among the five stages. The red, green, blue, light blue, and pink columns indicate stages 1 to 5, respectively. The number at the bottom indicates the cell line number. Color scale indicates number of standard deviations from the overall average of the lipid species with the areas in red indicating a higher amount of the lipid species, whereas the areas in blue indicating a lower amount of the lipid species. (b) Scatterplots of p-values obtained using the one-way analysis of variance of 309 lipid species among the five stages. Straight line indicates p-value of 0.05. Red dots show statistically significant lipids (false discovery rate < 0.05). Both figures were generated using MetaboAnalyst 5.0.

We used three cell lines (Cell Line-1, Cell Line-2, and Cell Line-3) of Caucasian-derived subcutaneous preadipocytes. Each cell line was divided into five stages: subcutaneous preadipocytes (stage-1), after inducing differentiation into adipocytes (stage-2), after the initiation of fat accumulation, the early stage (stage-3), the middle stage (stage-4), and mature subcutaneous adipocytes (stage-5), respectively. Lipid droplets in the three cell lines at each stage were stained with BODIPY (boron-dipyrromethene,Thermo Fisher Scientific, San Jose, CA, USA) in the Adipocyte Fluorescent Staining Kit (PMC, Hokkaido, Japan), and nuclei were stained with Hoechst 33342 solution (2′-(4-Ethoxyphenyl)-5-(4-methyl-1-piperazinyl)-2,5′-bi-1H-benzimidazole, trihydrochloride, DOJINDO, Japan) are shown in the Supplemental Fig. 1. Lipids were extracted from the cells at each stage by employing the Bligh and Dyer method [1] and processed using untargeted liquid chromatography Q-Exactive Orbitrap tandem mass spectrometry (LC-MS/MS). The data were acquired using Xcalibur 4.3.73.11 (Thermo Scientific). Total ion chromatograms of the three cell lines, each cell line with the five stages, are shown in Fig. 1. All raw data were uploaded and processed using LipidSearch 4.2.13. Lipid nomenclatures are summarized in Table 1. Finally, all data were normalized and exported for bioinformatic analysis using MetaboAnalyst 5.0 (https://www.metaboanalyst.ca/) [2]. The lipids identified using LipidSearch 4.2.13 and the normalized area values are given in Supplemental Table S1. Dendrogram and principal component analysis clearly separated different stages of cells such as subcutaneous preadipocytes (stage-1), after the induction of differentiation into adipocytes (stage-2), and after the start of fat accumulation (stage-3 to stage-5) are shown in Fig. 2. The heatmaps of the lipid species and scatterplots of p-values of one-way analysis of variance (ANOVA) are shown in Fig. 3. Of the 309 lipid species detected using LipidSearch 4.2.13, 145 (6 LPCs, 7 PCs, 2 LPEs, 25 PEs, 2 PSs, 7 PGs, 6 PIs, 14 DGs, 73 TGs, and 3 ChEs) were statistically significant (false discovery rate, (FDR) < 0.05) and are listed in Supplemental Table S2. The box and whisker plots of the top 50 lipid species that were statistically significant (FDR < 0.05) are shown in Supplemental Fig. 2. The number of lipid species detected using LipidSearch 4.2.13 and the number of statistically significant lipid species (FDR < 0.05) are summarized in Table 2.

**Table 1:** LipidSearch nomenclature of the identified lipid species

Group	ClassKey	Lipid name
P-Choline	LPC	Lysophosphatidylcholine
	PC	Phosphatidylcholine
P-Ethanol amine	LPE	lysophosphatidylethanolamine
	PE	phosphatidylethanolamine
P-Serine	PS	Phosphatidylserine
P-Glycerol	PG	Phosphatidylglycerol
P-Inositol	PI	Phosphatidylinositol
P-Acid	PA	phosphatidic acid
Sphingolipids	SM	Sphingomyelin
Neutral glycerolipid	DG	Diacylglycerol
	TG	Triacylglycerol
Steroid	ChE	Cholesterol ester

**Table 2:** Summary of the number of lipids detected and the number of statistically significant lipids

Class	Lipids detected	Statistically significant lipids(FDR <0.05)
LPC	34	6
PC	31	7
LPE	7	2
PE	70	25
PS	16	2
PG	11	7
PI	10	6
PA	0	0
SM	4	0
DG	20	14
TG	100	73
ChE	6	3
Total	309	145

The one-way analysis of variance was performed using MetaboAnalyst 5.0 to identify statistically significant lipids. Abbreviations: LPC, lysophosphatidylcholine; PC, phosphatidylcholine; LPE, lysophosphatidylethanolamine; PE, phosphatidylethanolamine; PS, phosphatidylserine; PG, phosphatidylglycerol; PI, phosphatidylinositol; PA, phosphatidic acid; SM, sphingomyelin; DG, diacylglycerol; TG, triacylglycerol; ChE, cholesterol ester; FDR, false discovery rate.

## 2 Experimental Design, Materials and Methods

### 2.1 Cell culture

Three cell lines of primary cultured human preadipocytes were used for the experiments; Cell Line-1 and Cell Line-2 were provided by Promocell (GmbH, Heidelberg, Germany) and Cell Line-3 by Zen-Bio (Zen-Bio, Inc., Research Triangle Park, NC, USA). All the three lines were obtained from subcutaneous adipose tissues from the abdomen of Caucasian women with a mean age of 51.3 ± 5 years. These preadipocytes were seeded into a 12-well plate; their growth and in-vitro differentiation into adipocytes were performed according to the manufacturer's recommendations. Briefly, the preadipocytes were grown at 37°C with 5% CO_2_ in a humidified incubator in Promocell Preadipocyte Growth Medium containing 5% fetal calf serum, 0.4% endothelial cell growth supplement, 10 ng/mL human recombinant epidermal growth factor, 1 μg/mL hydrocortisone, and 90 μg/mL heparin until 100% confluent growth was observed (stage-1). Further, their differentiation into adipocytes was induced using Promocell Preadipocyte Differentiation Medium containing 8 μg/mL biotin, 0.5 μg/mL human recombinant insulin, 400 ng/mL dexamethasone, 44 μg/mL 3-isobutyl-1-methylxanthine, 9 ng/mL thyroxine, and 3 μg/mL ciglitazone for 3 consecutive days. The post-differentiation adipocytes were designated as stage-2. After the induction of differentiation, Promocell Preadipocyte Differentiation Medium was replaced with Promocell Adipocyte Nutrition Medium containing 3% fetal calf serum, 8 μg/mL biotin, 0.5 μg/mL insulin, and 400 ng/mL dexamethasone. The culture was continued for 7 days until the cytoplasm was filled with many lipid droplets, and the old medium was replaced with fresh Promocell Adipocyte Nutrition Medium after every 2 to 3 days during culturing. During this culture period, small lipid droplets began to appear in the cytoplasm (stage -3), followed by more lipid droplets (stage-4) and the cytoplasm filled with many lipid droplets (stage-5).

### 2.2 Fluorescent staining and microscopy

Lipid droplets in each stage of the three cell lines were stained with BODIPY (boron-dipyrromethene, Thermo Fisher Scientific) in the Adipocyte Fluorescent Staining kit (PMC), and nuclei were stained with Hoechst 33342 solution (2′-(4-Ethoxyphenyl)-5-(4-methyl-1-piperazinyl)-2,5′-bi-1H-benzimidazole, trihydrochloride, DOJINDO). The cells were washed once with washing buffer, and fixed with 10% formalin neutral buffer solution (Wako, Osaka, Japan) at room temperature for overnight. The formalin neutral buffer solution was removed, and the cells were washed twice with washing buffer. The cells were incubated with BODIPY for 30 min at room temperature, and then BODIPY was removed, and the cells were washed once with wash buffer. In addition, the cells were incubated with Hoechst 33342 for 30 min at room temperature, then Hoechst 33342 was removed, and the cells were washed once with wash buffer. Finally, the adipocytes were treated with the mounting agent. Cell images were observed using an Olympus IX-83 inverted fluorescence microscope (Olympus, Tokyo, Japan). The excitation/emission wavelengths were controlled using cellSens Dimension software version 1.12. Image analysis was performed using ImageJ Fiji Software (NIH, Bethesda, MD) [3], [4], [5], [6] (Supplemental Fig. 1).

### 2.3 Lipid extraction

The cultured Caucasian-derived subcutaneous adipocytes were washed with 2-[4-(2-hydroxyethyl)-1-piperazinyl]ethanesulfonic acid (HEPES) buffered balanced salt solution, treated with trypsin/EDTA (0.025%/0.01%) solution at 25°C, neutralized with trypsin neutralization solution. The cell suspension was transferred to a tube and centrifuged at 220*g* for 3 min, the supernatant was discarded and the cells were suspended in 100 μL of Milli-Q water. Each sample was normalized by measuring the amount of protein by bicinchoninic acid (BCA) protein assay using Pierce Micro BCA Protein Assay Kit (Pierce, Rockford, IL, USA) and the final volume of 600 μL was adjusted using water. The Bligh and Dyer method was used for lipid extraction. The cell suspension samples were transferred into glass tubes, and 1.5 mL of methanol was added, vortexed, and spiked with 10 μL of Splash Lipidomix internal standards (Avanti, Alabaster, AL, USA). Sequentially, 750 μL of chloroform was added, vortexed, and extracted for 10 min at 25°C. After the extraction, 750 µL of chloroform was added and the solution was vortexed, followed by the addition of 750 µL of distilled water and subsequent vortexing. The extracted samples were centrifuged at 1000*g* for 10 min. The upper layer (containing water and methanol) was discarded; the extracted organic layers (approximately 1.2 mL) were transferred into new glass tubes and evaporated to achieve complete drying using miVac Duo LV (Genevac, Ipswich, England). The extracted lipids were resolved with 150 μL of LC-MS–grade methanol.

### 2.4 High-performance liquid chromatography (HPLC) and tandem mass spectrometry

The extracted lipids from the cultured Caucasian-derived subcutaneous adipocytes were analyzed using Q-Exactive Hybrid Quadrupole-Orbitrap Mass Spectrometer equipped with an electrospray ionization source and connected to the Ultimate 3000 HPLC system (Thermo Scientific). The extracted lipid samples (10 μL) were injected and separated using the Acclaim 120 C18 column (3 μm, 2.1 mm × 150 mm) (Thermo Scientific). The temperature of the column was maintained at 50°C and that of the tray was maintained at 10°C. Solvent A was composed of 5 mM ammonium formate at 2:1:1 (water:methanol:acetonitrile) with 0.1% formic acid. Solvent B was composed of 5 mM ammonium formate at 9:1 (isopropanol: acetonitrile) with 0.1% formic acid. The flow rate was 300 μL/min. All solvents and reagents used for HPLC were purchased from Fujifilm Wako Pure Chemical Industries (Osaka, Japan) and were of LC-MS grade. We used a set of linear gradients starting at 20% of solvent B and increasing linearly to 100% over 50 min, maintained at 100% solvent B for 60 min, then decreased linearly to 20% B from 60 min to 60.1 min, and finished with 20% solvent B for the last 10 min. The mass spectrometer was operated under heated electrospray ionization in positive and negative modes. The spray voltage was 3.5 kV (positive mode) or 2.5 kV (negative mode) and the capillary temperature was 250°C. The S-lens radiofrequency was 50 Hz. The sheath gas flow rate was 50 (arbitrary units) and the auxiliary gas flow rate was 15 (arbitrary units). The full scan used a resolution of 70,000 with an automatic gain control (AGC) target of 1 × 10^6^ ions and a maximum ion injection time (IT) of 100 ms. Data-dependent MS/MS (top 5) was performed using the following parameters: resolution, 17,500; AGC, 1 × 10^5^; maximum IT, 80 ms; isolation window, 2.0 m/z; underfill ratio, 4.0%; intensity threshold, 5 × 10^4^; and dynamic exclusion time, 8.0 s. Normalized collision energy settings were 25.5, 30.0, and 34.5 (positive mode) or 19.5, 30.0, and 40.5 (negative mode).

### 2.5 Lipid identification and statistical analysis

All LC-MS/MS data files were processed using LipidSearch 4.2.13 to identify the lipid molecular species within each lipid fraction. The settings of LipidSearch were as follows: precursor tolerance, 5 ppm; product tolerance, 8 ppm; m-score threshold, 2; Quan m/z tolerance ± 5 ppm; Quan retention time (RT) range ± 0.5 min; use of main isomer filter and for the ID quality filter, A-B; adduct ions, H^+^ and NH_4_^+^ for positive ion mode and H^−^ and HCOO^−^ for negative ion mode. The lipid classes selected for the search were lysophosphatidylcholine (LPC), phosphatidylcholine (PC), lysophosphatidylethanolamine (LPE), phosphatidylethanolamine (PE), phosphatidylserine (PS), phosphatidylglycerol (PG), phosphatidylinositol (PI), phosphatidic acid (PA), sphingomyelin (SM), diacylglycerol (DG), triacylglycerol (TG), and cholesterol ester (ChE). For data alignment, the ID quality filters were A and B, the RT was 1.0 min, and the top-ranked filtered and main isomer peaks were selected. The output file was processed by normalizing the raw peak areas with the values of the areas of their corresponding lipid class internal standard (18:1(d7) LPC, 15:0-18:1(d7) PC, 18:1(d7) LPE, 15:0-18:1(d7) PE, 15:0-18:1(d7) PS, 15:0-18:1(d7) PG, 15:0-18:1(d7) PI, 15:0-18:1(d7) PA, d18:1-18:1(d9) SM, 15:0-18:1(d7) DG, 15:0-18:1(d7)-15:0 TG, and 18:1(d7) ChE) and formatted for further statistical analysis using MetaboAnalyst 5.0. The data were log-transformed, scaled by mean-centering, and divided by the standard deviation of each variable (autoscaling). Data were analyzed using the one-way ANOVA followed by Fisher's least significant difference (LSD) post hoc tests (FDR < 0.05).

## Ethics Statements

The authors declare that this work does not involve the use of human subjects, social media data, or experimentation with animals.

## CRediT Author Statement

**Aya Kitamoto:** Conceptualization, Methodology, Software, Validation, Formal analysis, Writing – review & editing; **Takuya Kitamoto:**Conceptualization, Methodology, Software, Investigation, Formal analysis, Writing – original draft preparation.

## Declaration of Competing Interest

The authors declare that they have no known competing financial interests or personal relationships that could have appeared to influence the work reported in this paper.

## Data Availability

Data on changes in lipid profiles during differentiation and maturation of human subcutaneous white adipocytes analyzed using chromatographic and bioinformatics tools (Original data) (Metabolomics Workbench).

## Appendix Supplementary materials

Image, application 1

Image, application 2

Image, application 3

Image, application 4
