# Supplementary material for: Data on changes in lipid profiles during the differentiation and maturation of human subcutaneous white adipocytes analyzed using chromatographic and bioinformatic tools
Source: Data Brief. 2022 May 6;42:108245. doi: 10.1016/j.dib.2022.108245 (PMC9114626; doi:10.1016/j.dib.2022.108245)

**Supplemental Figure 1.** Image analysis of three lines of Caucasian-derived subcutaneous adipocytes, five stages each. Images of the nuclei and lipid droplets were acquired using the filter set for DAPI (blue) and GFP (Green), respectively. Scale bars: 250 μm.

**Stage-1**

**Stage-2**

**Stage-3**

**Stage-4**

**Stage-5**


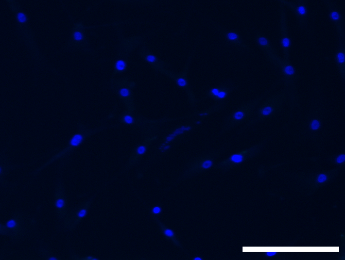


**Cell Line-1**


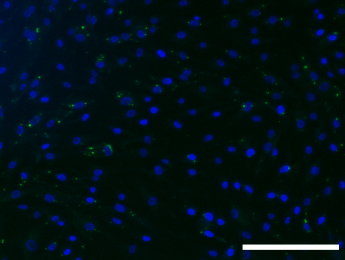

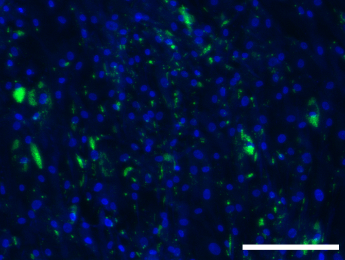

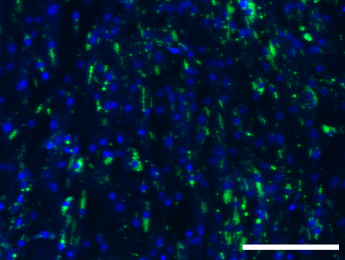

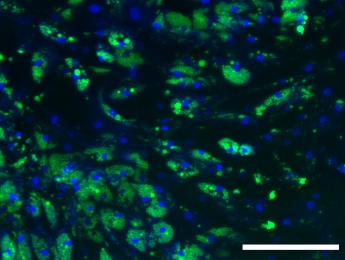


**Cell Line-2**


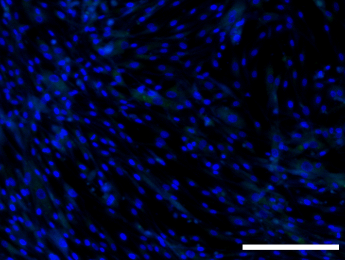

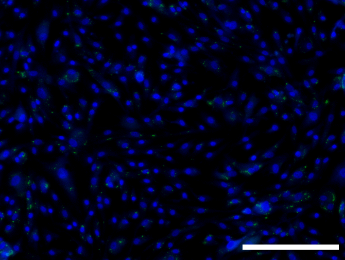

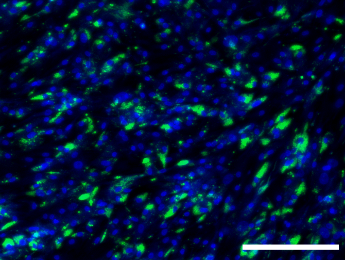

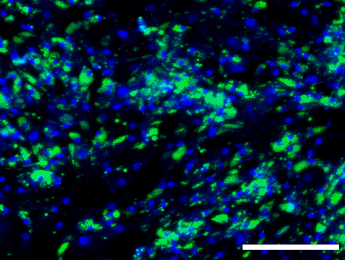

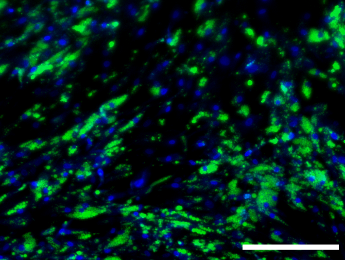


**Cell Line-3**


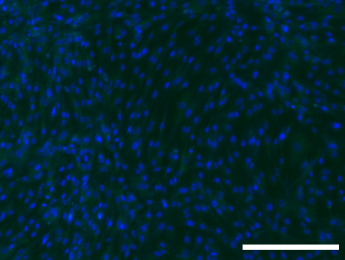

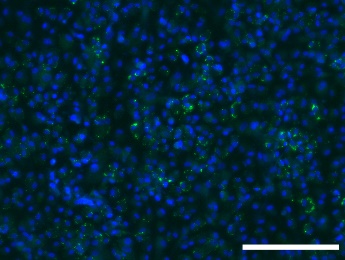

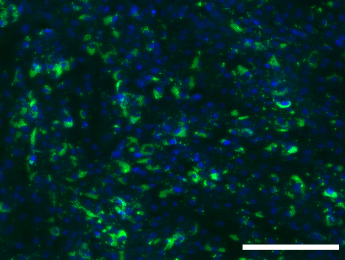

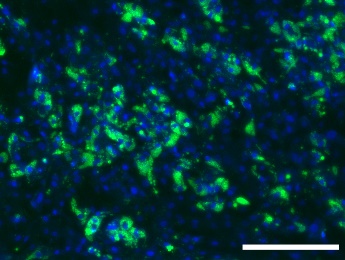

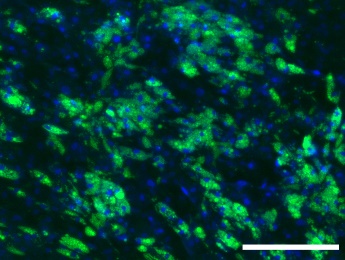

Supplement: Supplementary file 1 [file mmc1.docx]
