# Supplementary material for: Data on changes in lipid profiles during the differentiation and maturation of human subcutaneous white adipocytes analyzed using chromatographic and bioinformatic tools
Source: Data Brief. 2022 May 6;42:108245. doi: 10.1016/j.dib.2022.108245 (PMC9114626; doi:10.1016/j.dib.2022.108245)
Supplement: Supplementary file 2 [file mmc2.docx]

**Supplemental Figure 2.** Box and whisker plots of the top 50 lipid species that were statistically significant (false discovery rate, FDR < 0.05). The red, green, blue, light blue, and pink boxes indicate stages 1 to 5, respectively.


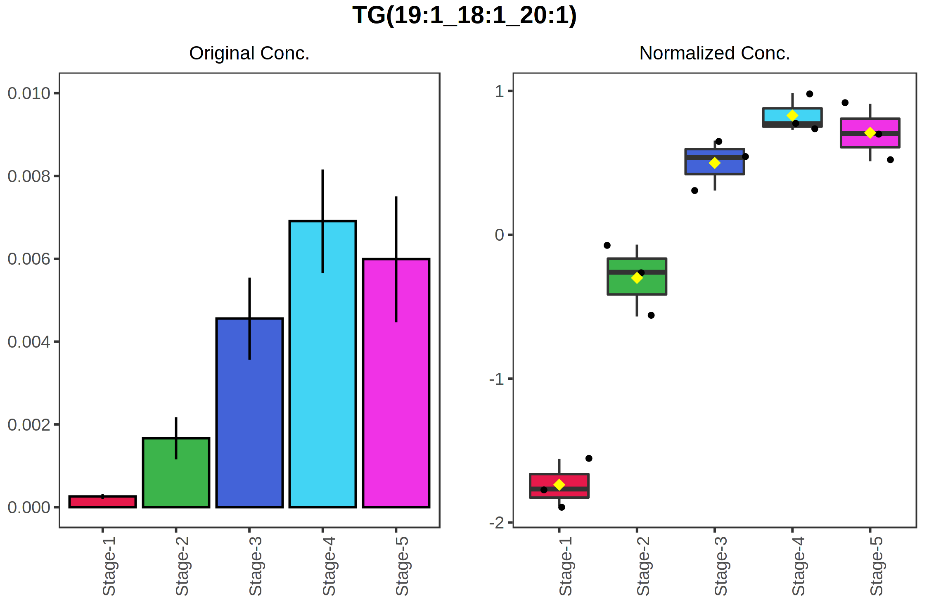

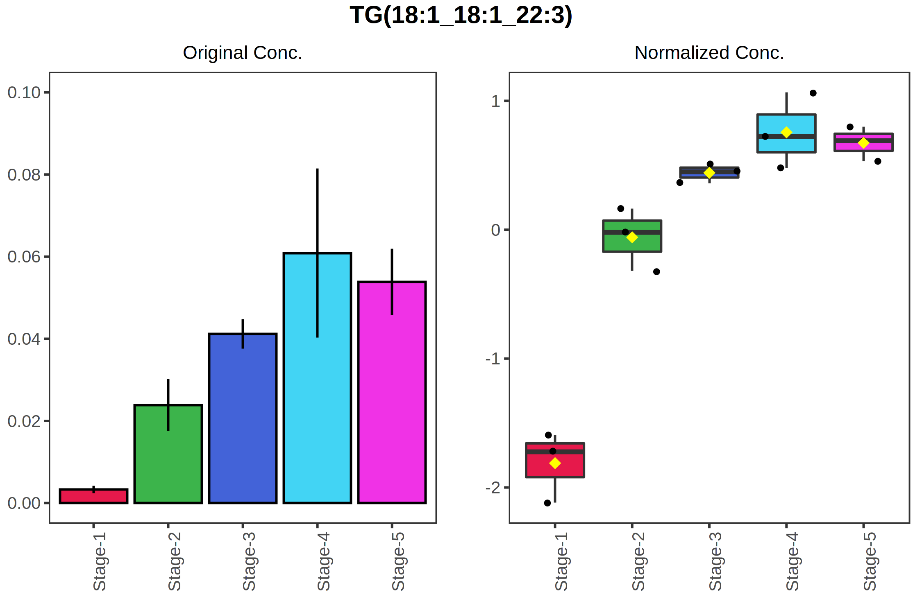

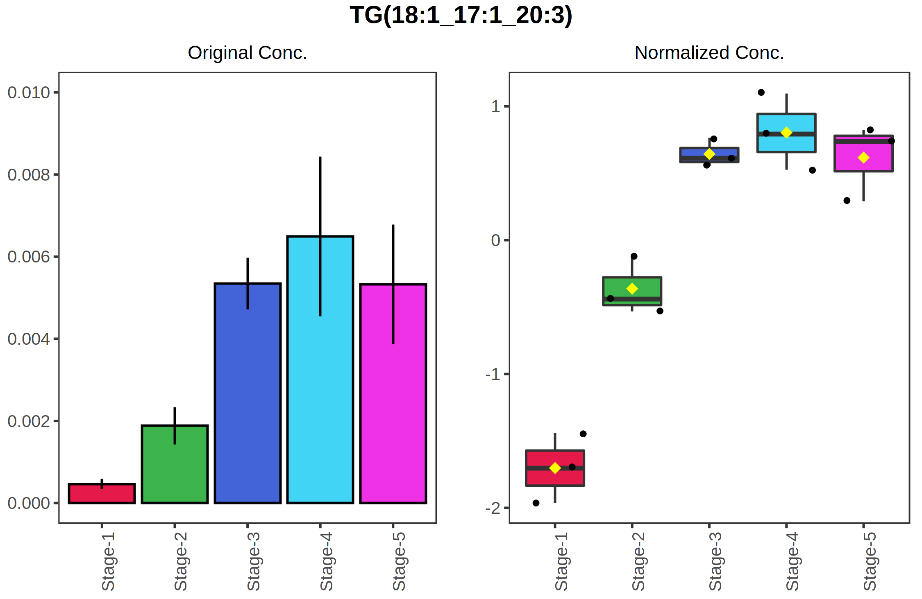

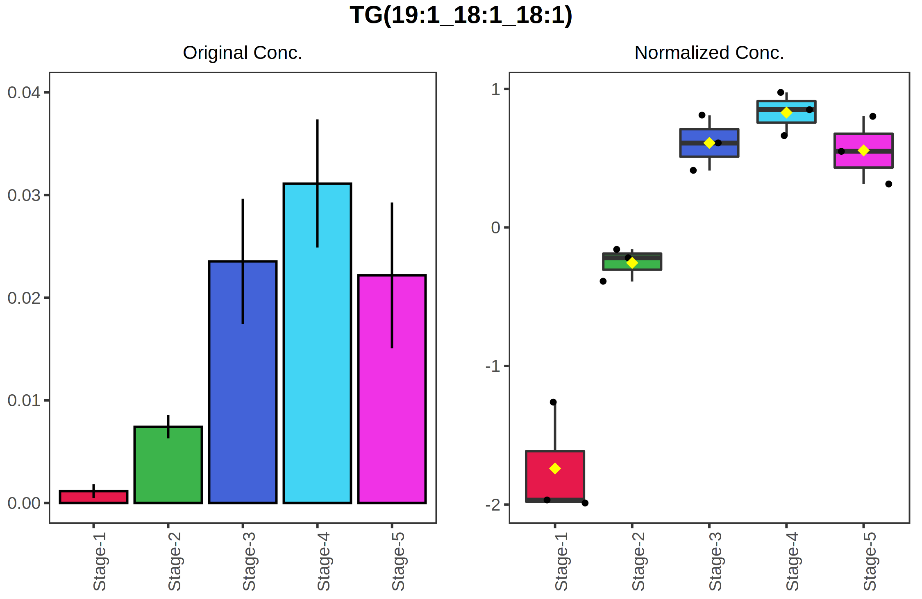

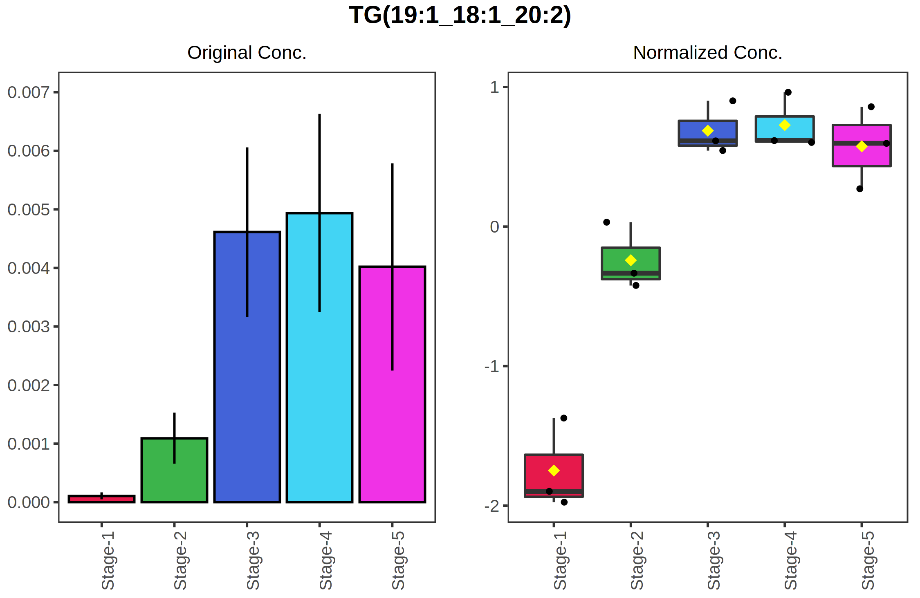

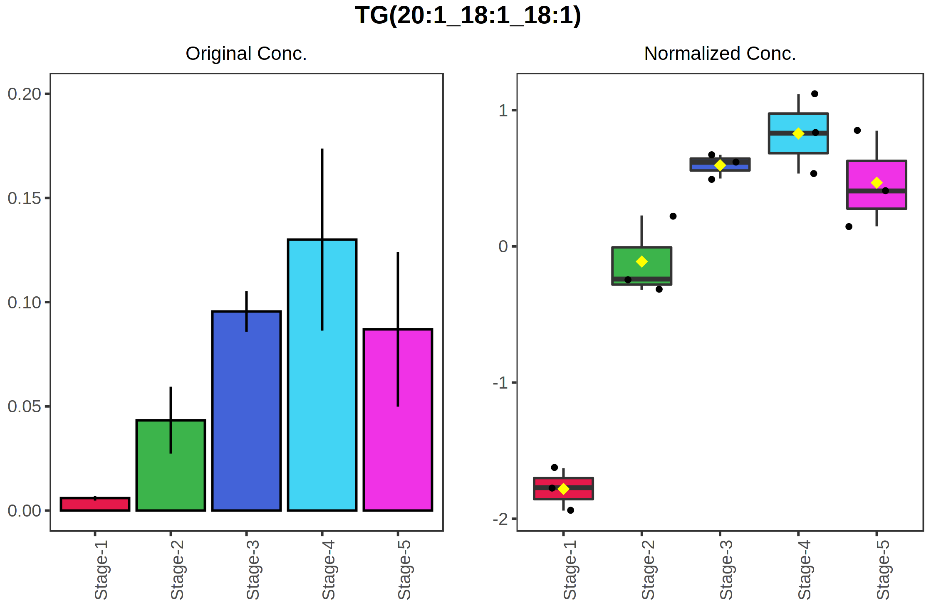

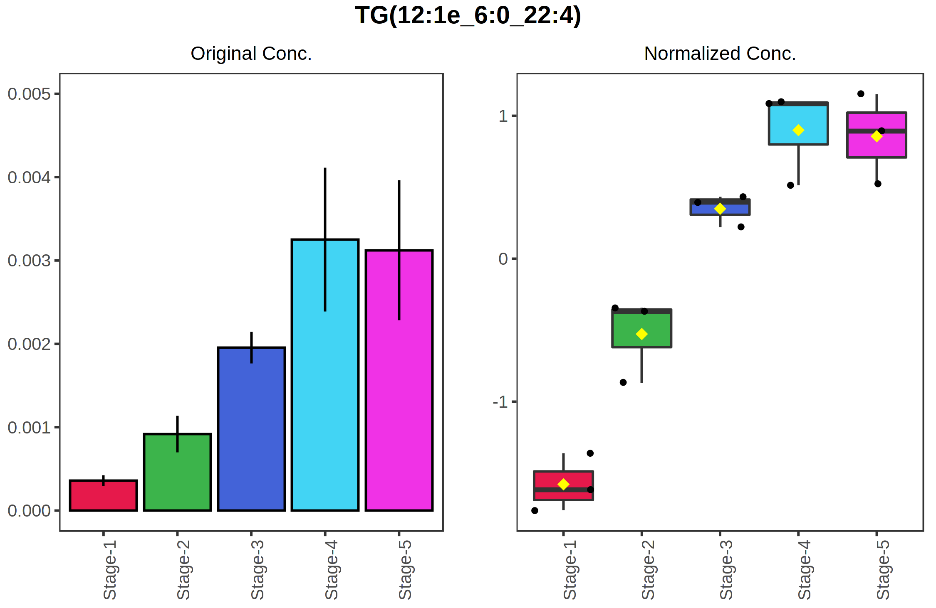

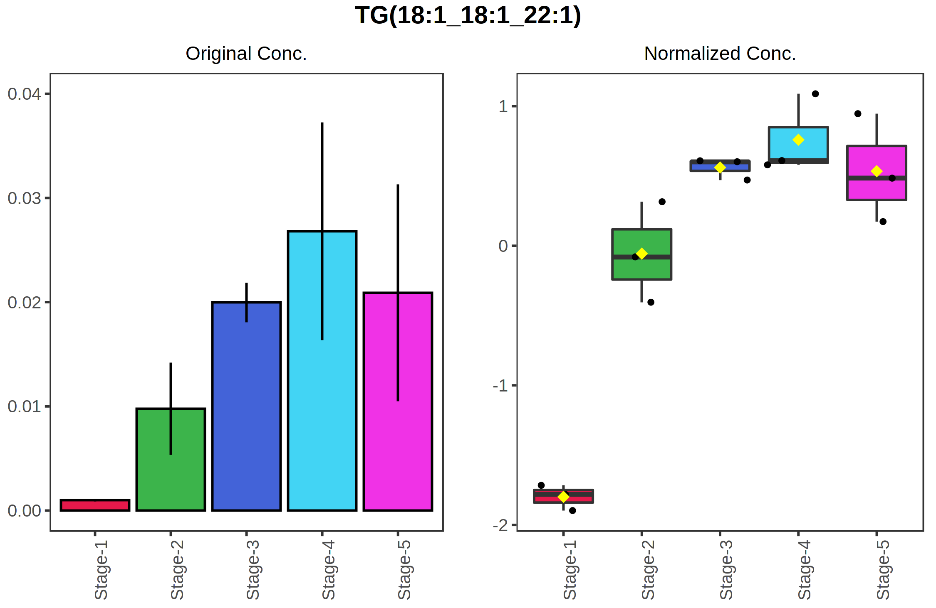

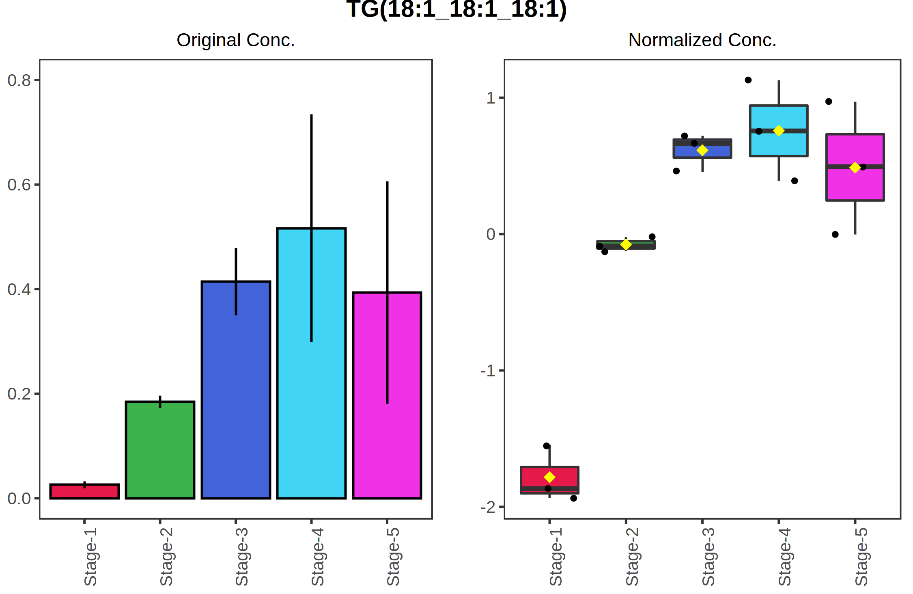

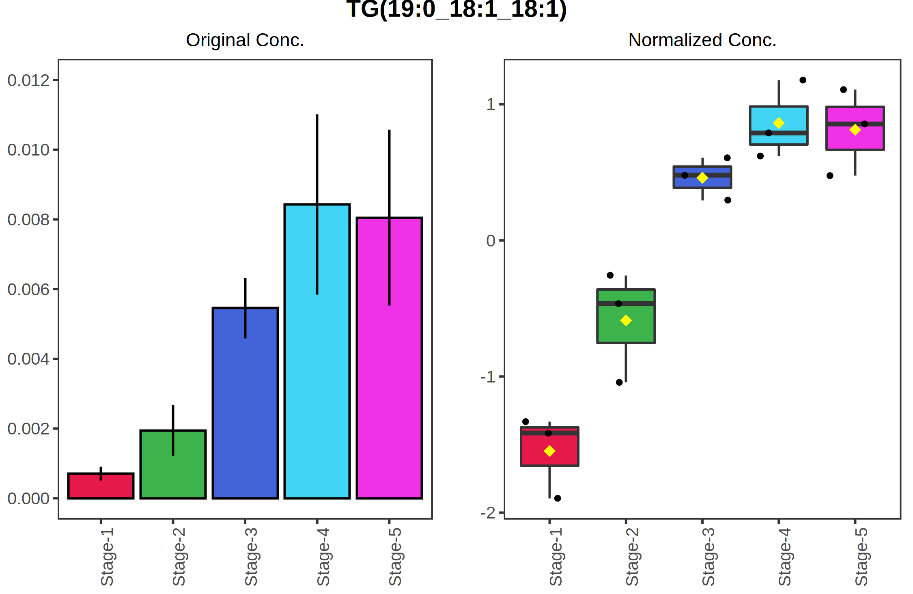


Normalized concentration

**TG (20:1_18:1_18:1)**

**TG (12:1e_6:0_22:4)**

**TG (18:1_18:1_22:1)**

**TG (18:1_18:1_18:1)**

**TG (19:0_18:1_18:1)**

**TG (19:1_18:1_20:1)**

**TG (18:1_18:1_22:3)**

**TG (18:1_17:1_20:3)**

**TG (19:1_18:1_18:1)**

**TG (19:1_18:1_20:2)**

Normalized concentration

Normalized concentration

Normalized concentration

Normalized concentration

Normalized concentration

Normalized concentration

Normalized concentration

Normalized concentration

Normalized concentration


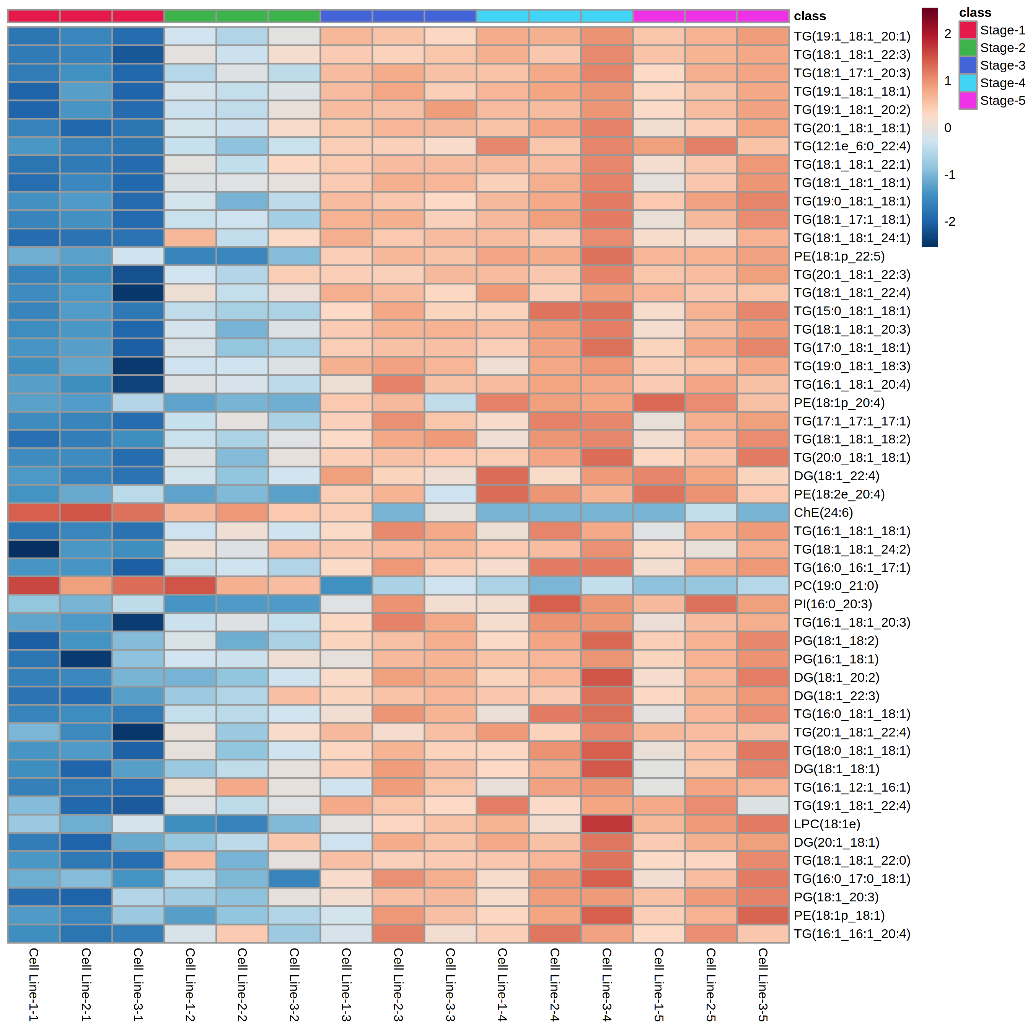


Stage-1

Stage-2

Stage-4

Stage-5

Stage-3


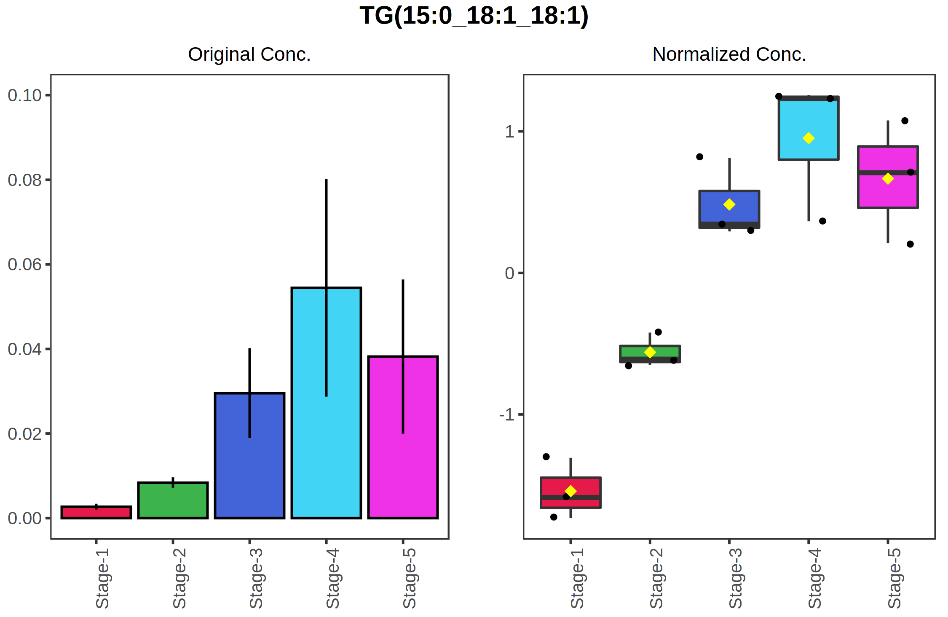

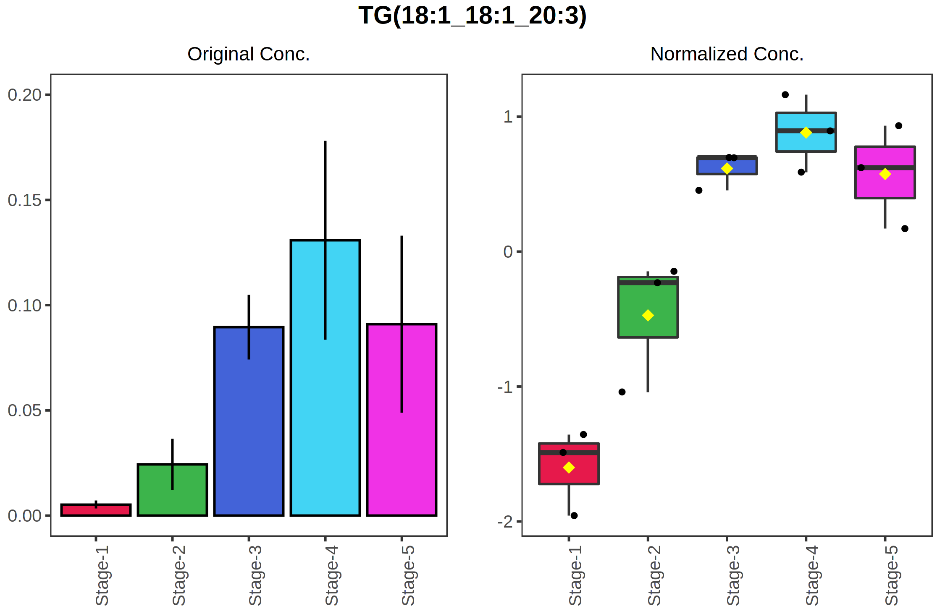

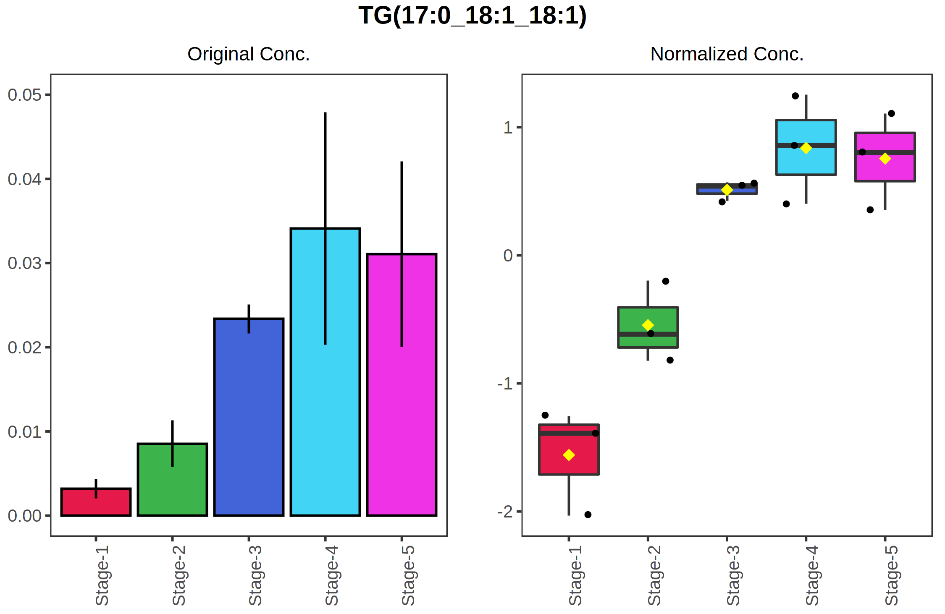

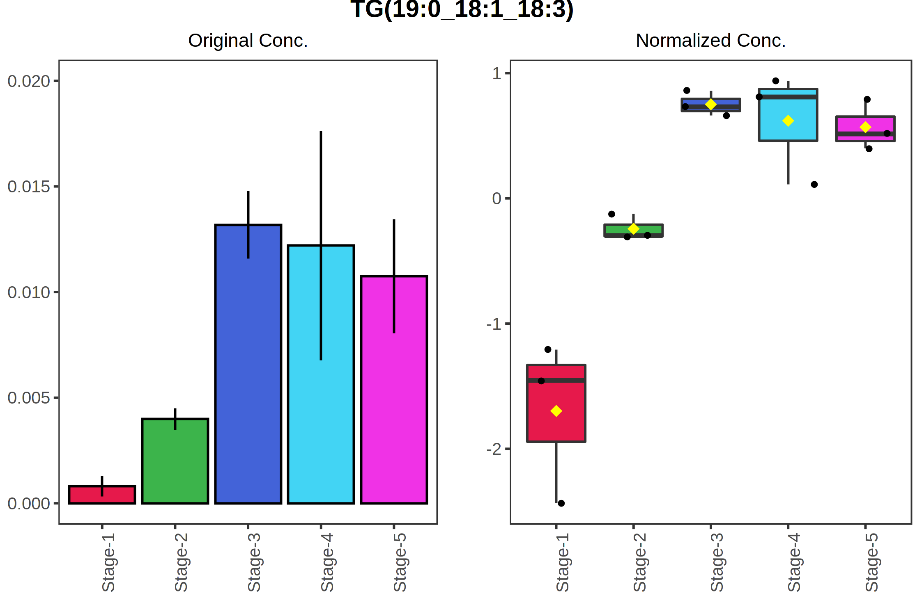

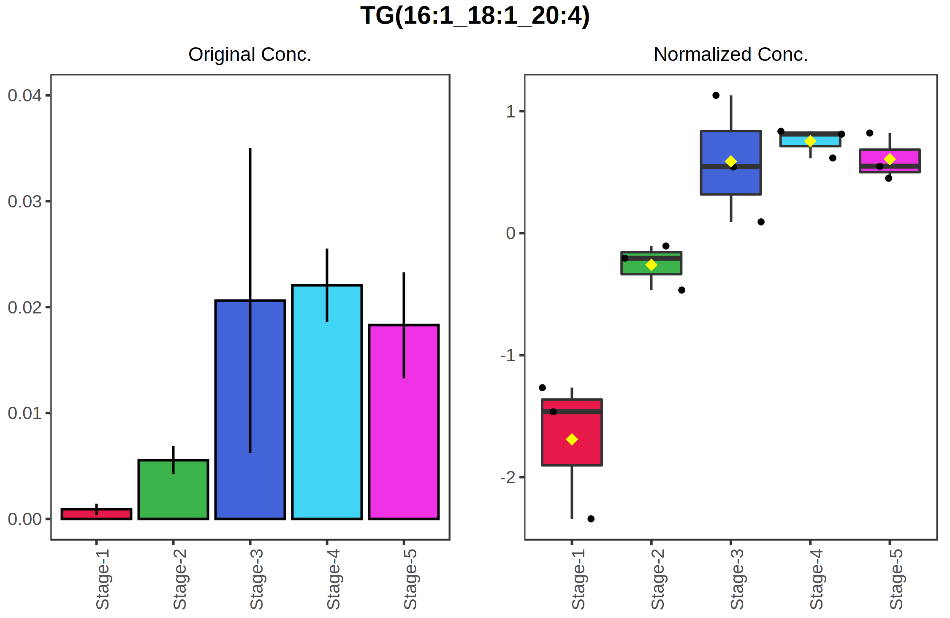


**TG (15:0_18:1_18:1)**

**TG (18:1_18:1_20:3)**

**TG (17:0_18:1_18:1)**

**TG (19:0_18:1_18:3)**

**TG (16:1_18:1_20:4)**

Normalized concentration

Normalized concentration

Normalized concentration

Normalized concentration

Normalized concentration


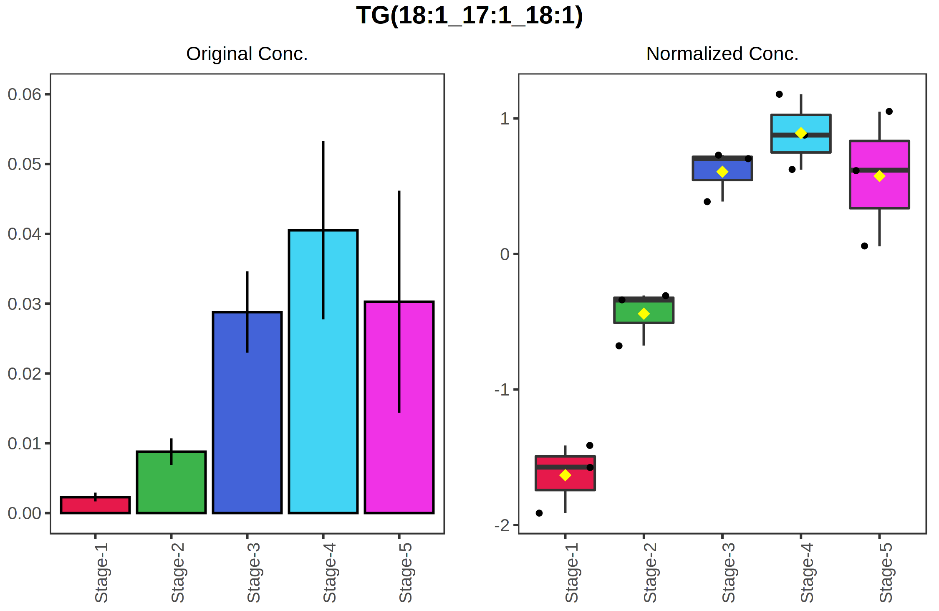

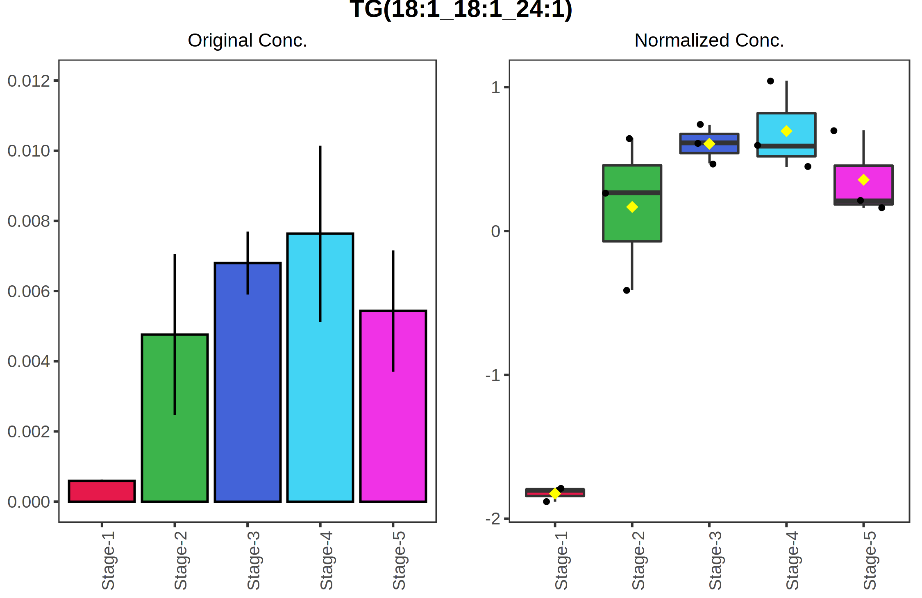

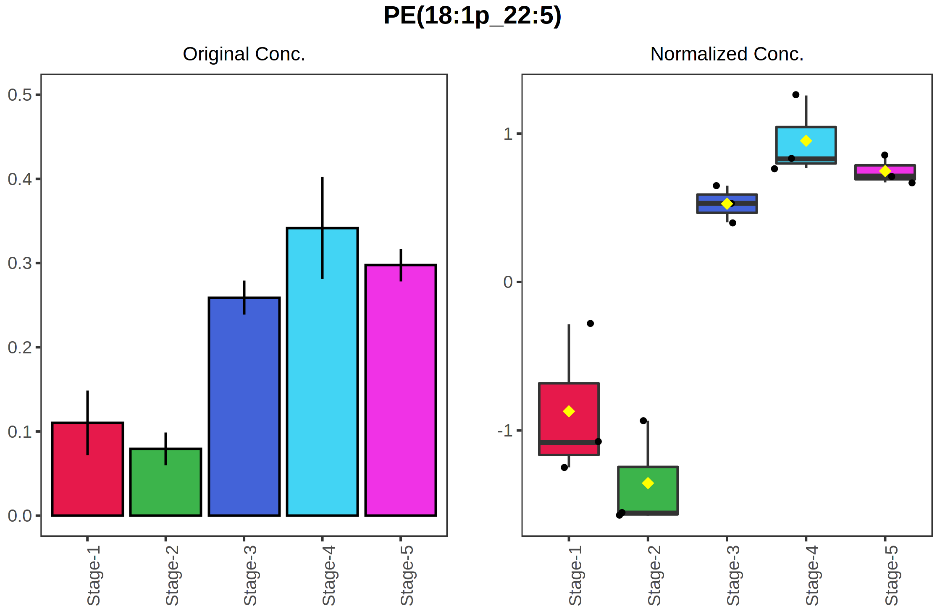

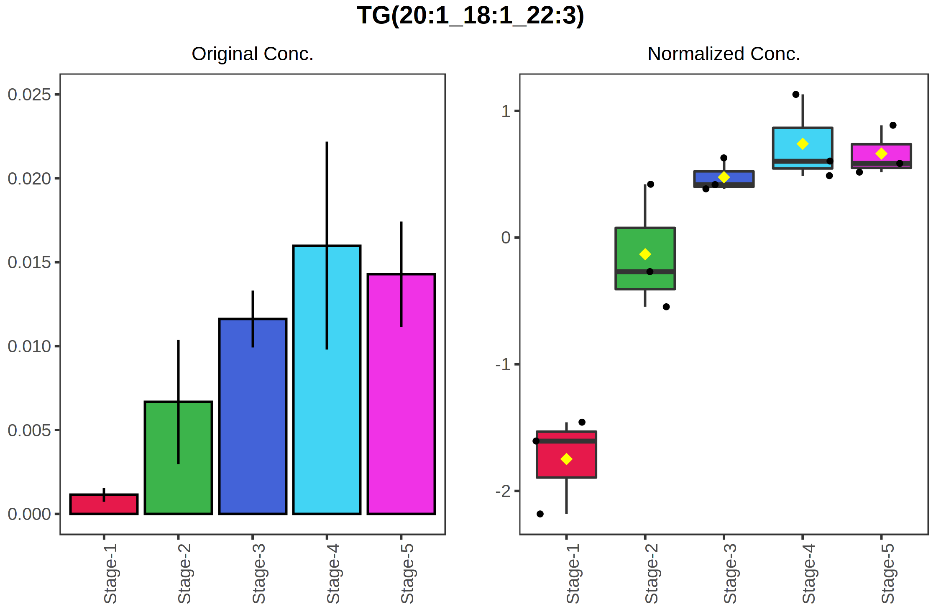

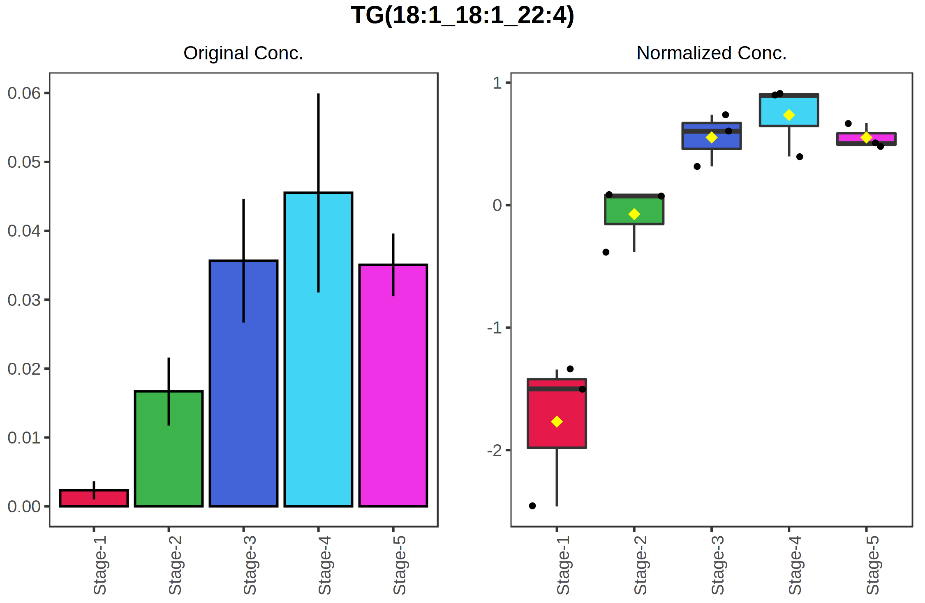


**TG (18:1_17:1_18:1)**

**TG (18:1_18:1_24:1)**

**PE (18:1p_22:5)**

**TG (20:1_18:1_22:3)**

**TG (18:1_18:1_22:4)**

Normalized concentration

Normalized concentration

Normalized concentration

Normalized concentration

Normalized concentration


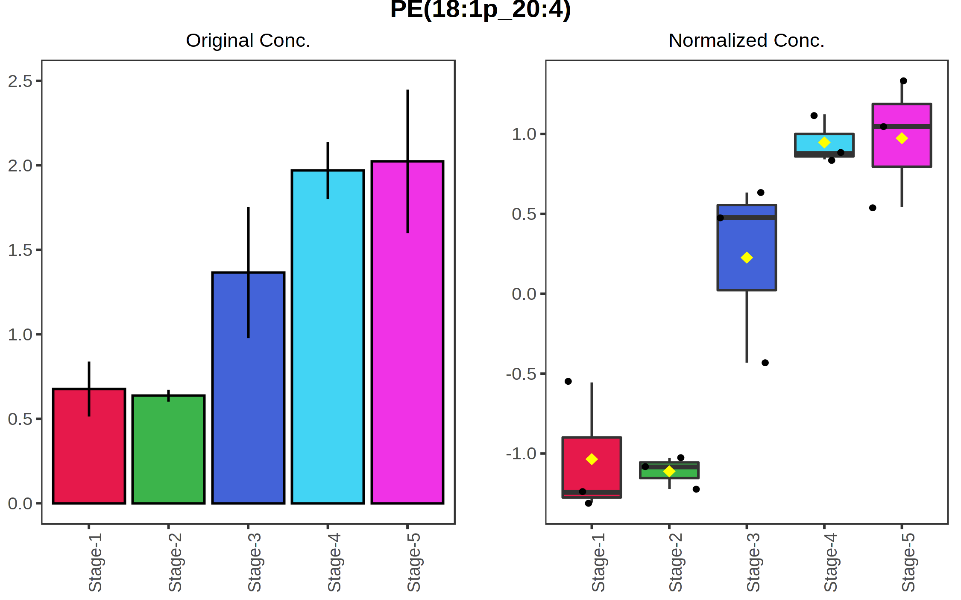

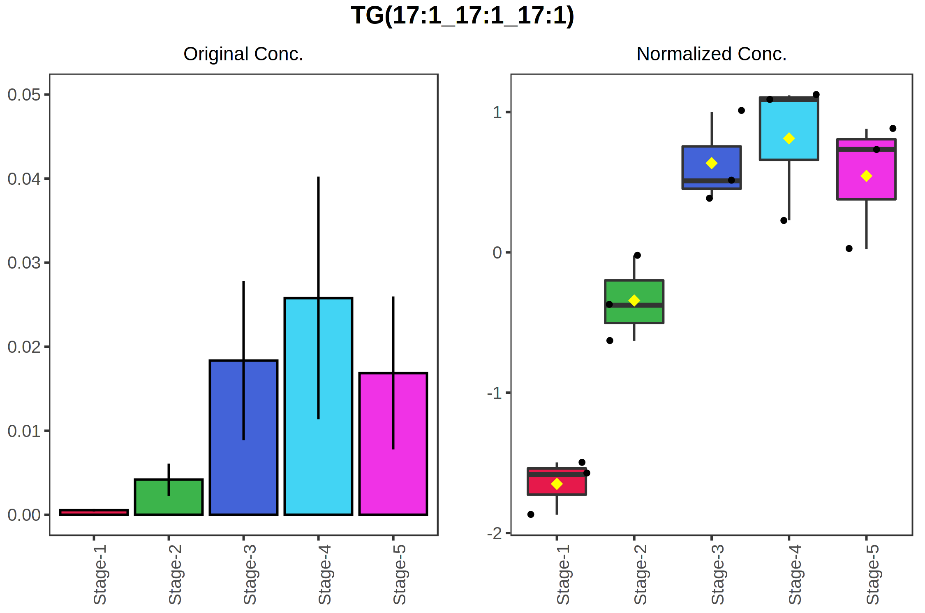

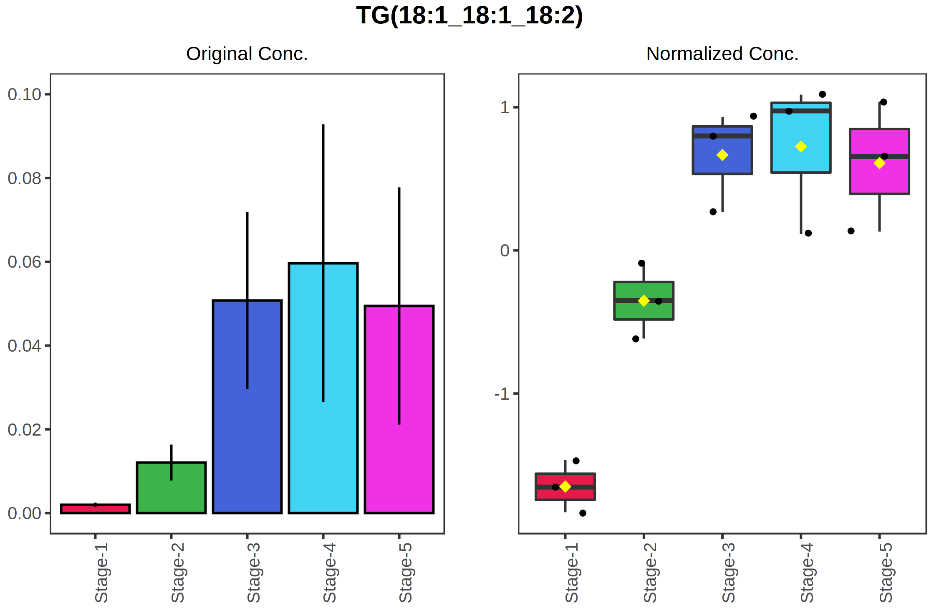

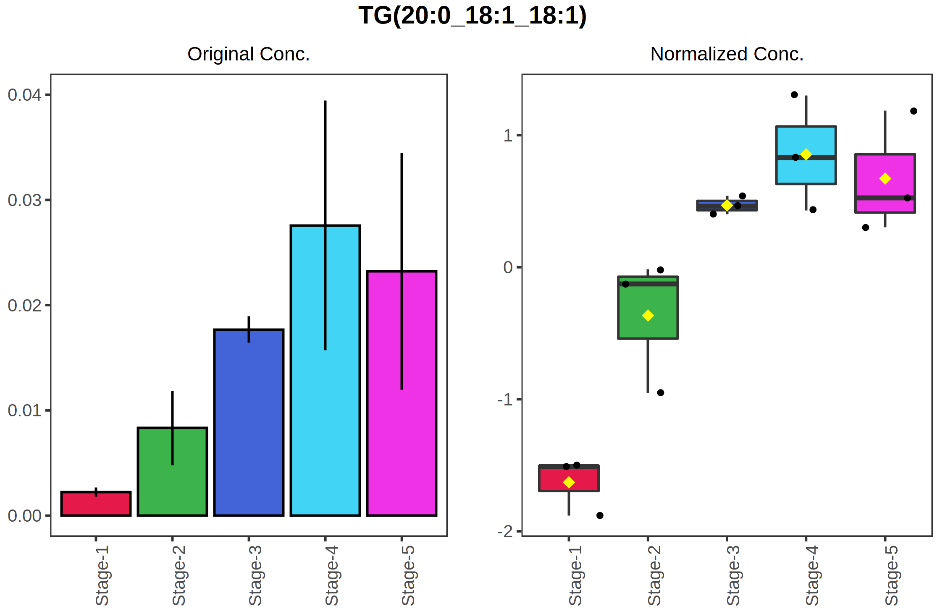

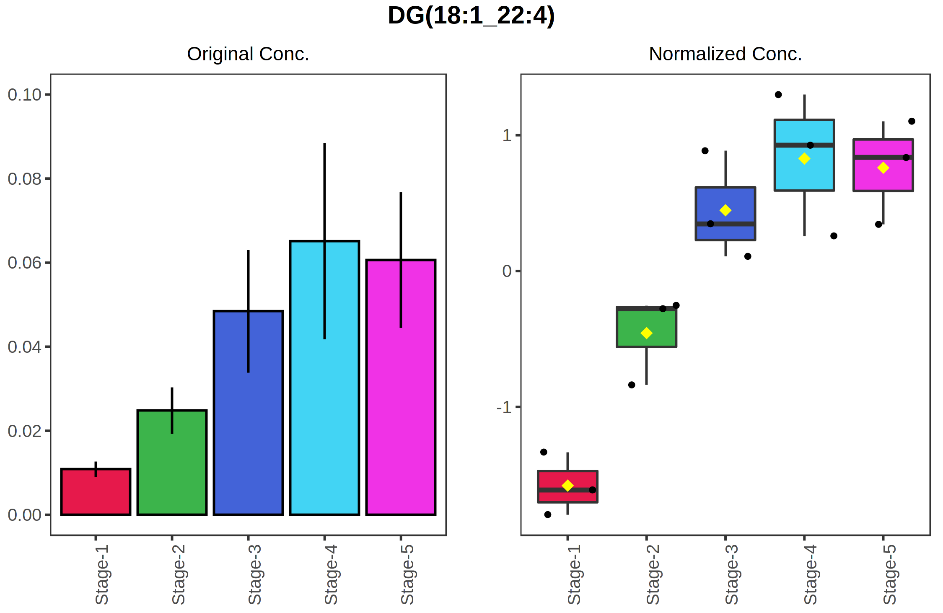


**PE (18:1p_20:4)**

**TG (17:1_17:1_17:1)**

**TG (18:1_18:1_18:2)**

**TG (20:0_18:1_18:1)**

**DG (18:1_22:4)**

Normalized concentration

Normalized concentration

Normalized concentration

Normalized concentration

Normalized concentration


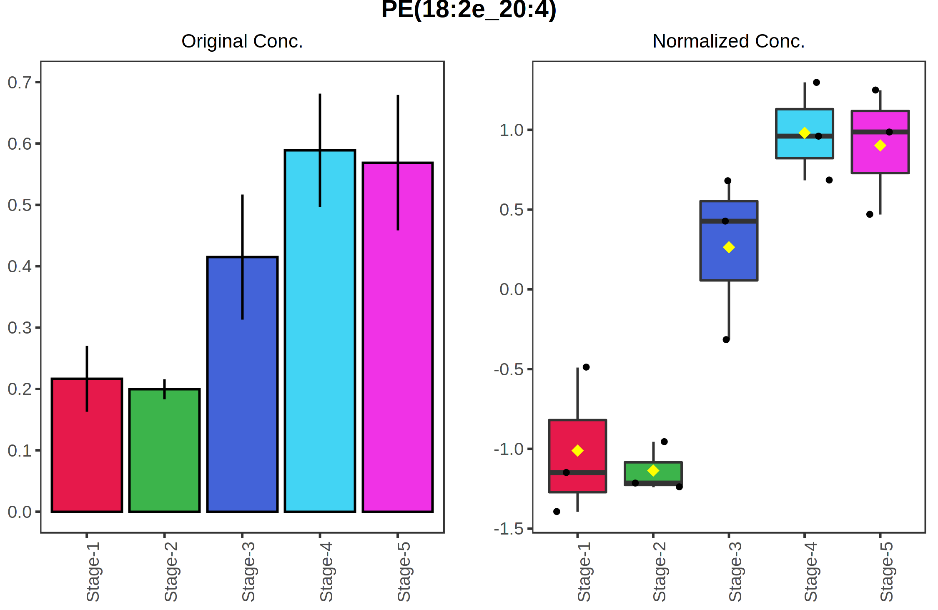

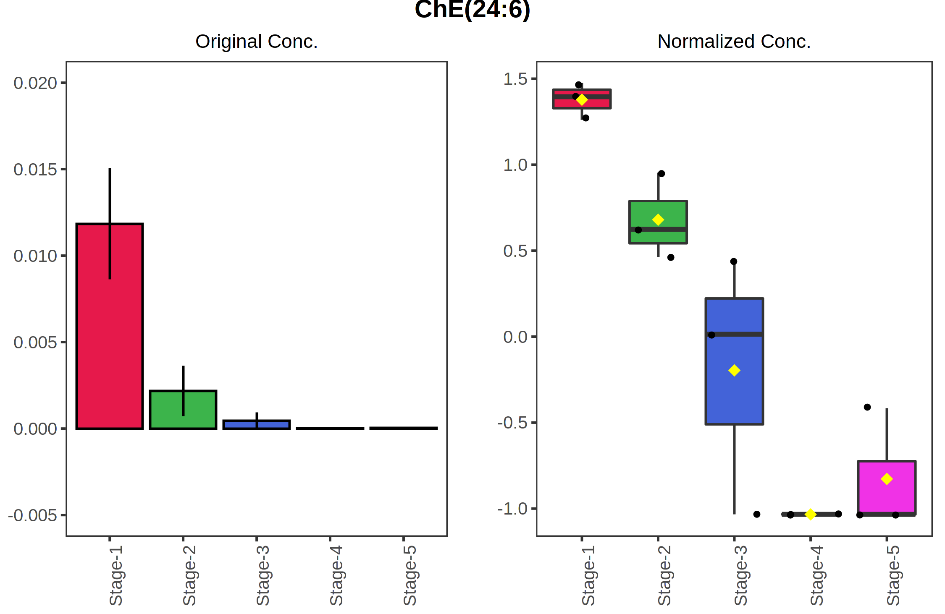

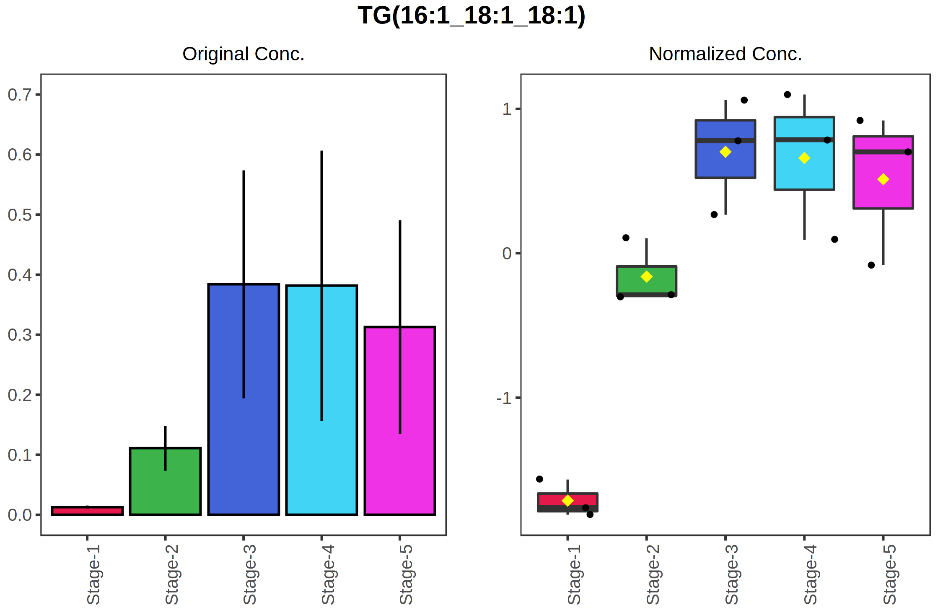

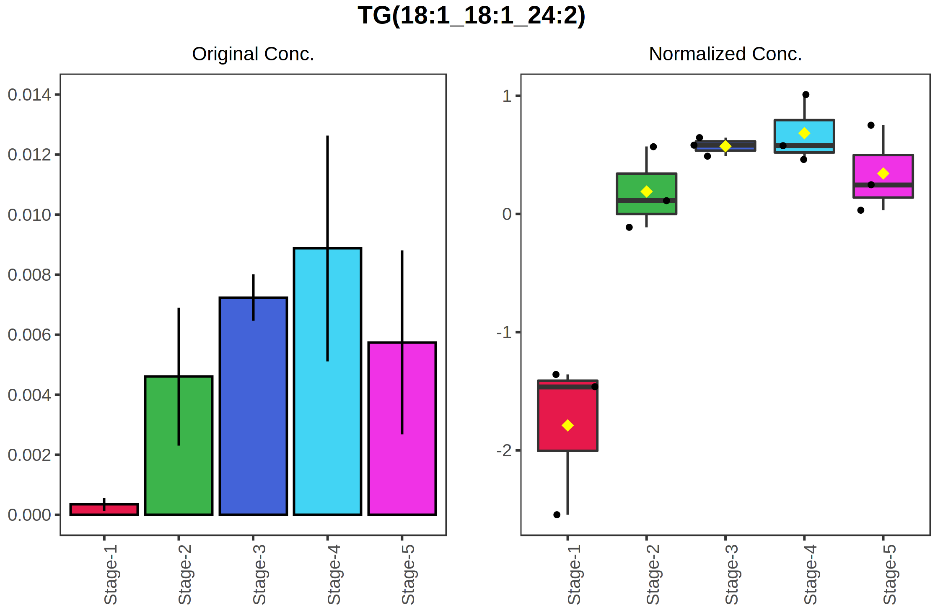

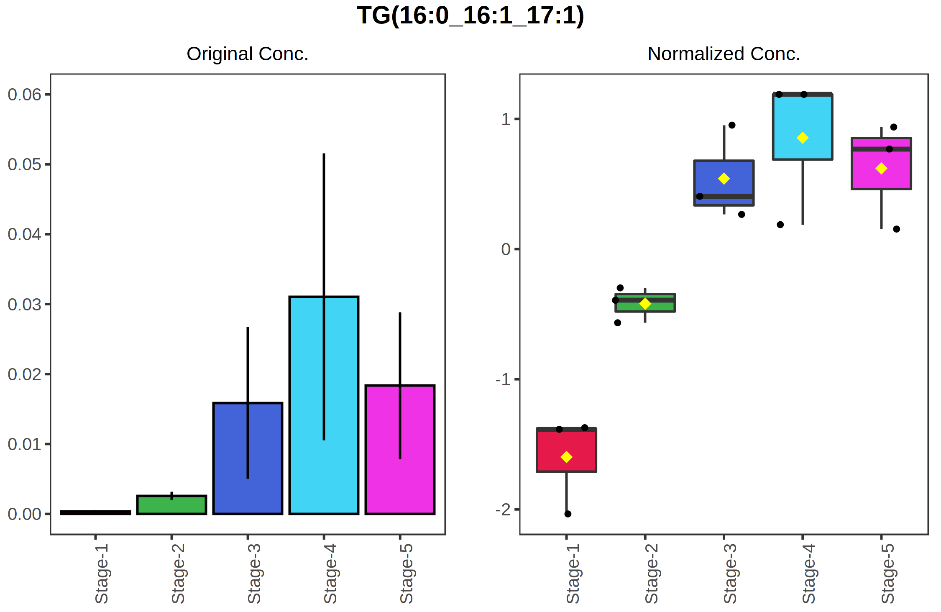


**PE (18:2e_20:4)**

**ChE (24:6)**

**TG (16:1_18:1_18:1)**

**TG (18:1_18:1_24:2)**

**TG (16:0_16:1_17:1)**

Normalized concentration

Normalized concentration

Normalized concentration

Normalized concentration

Normalized concentration


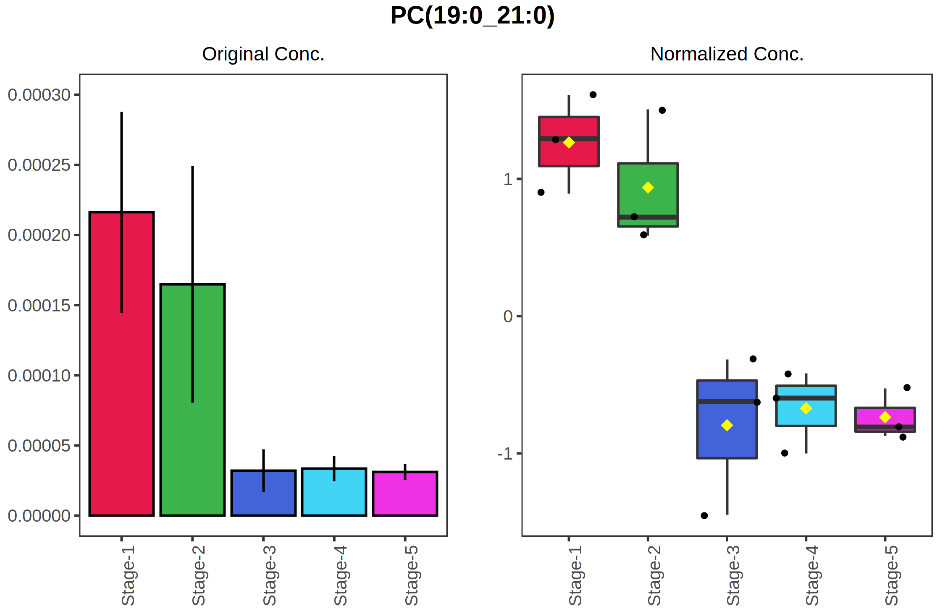

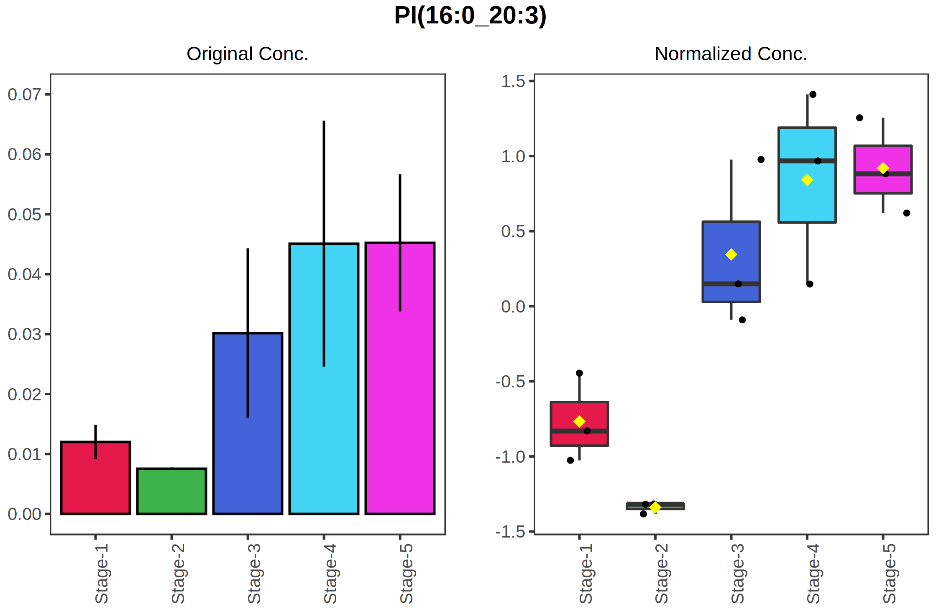

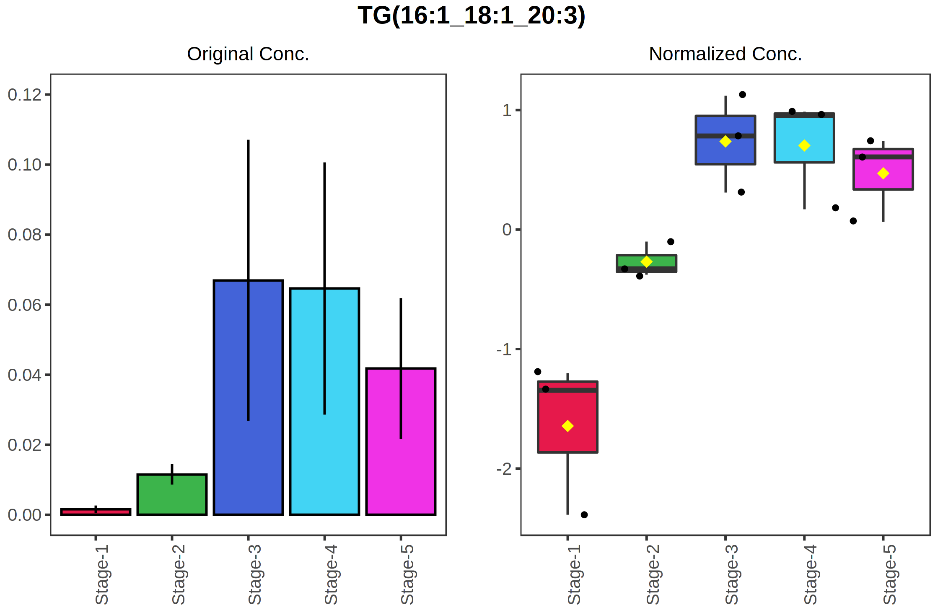

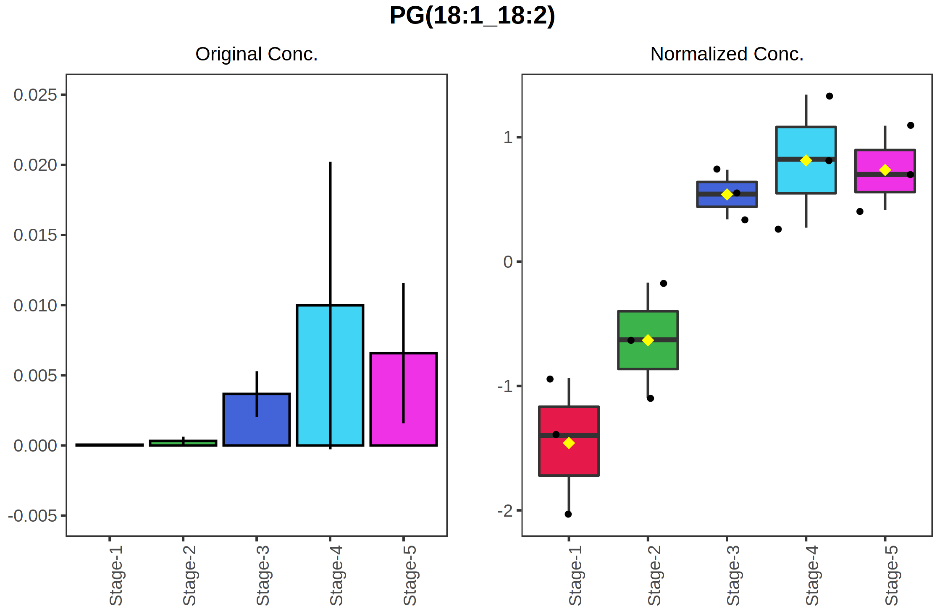

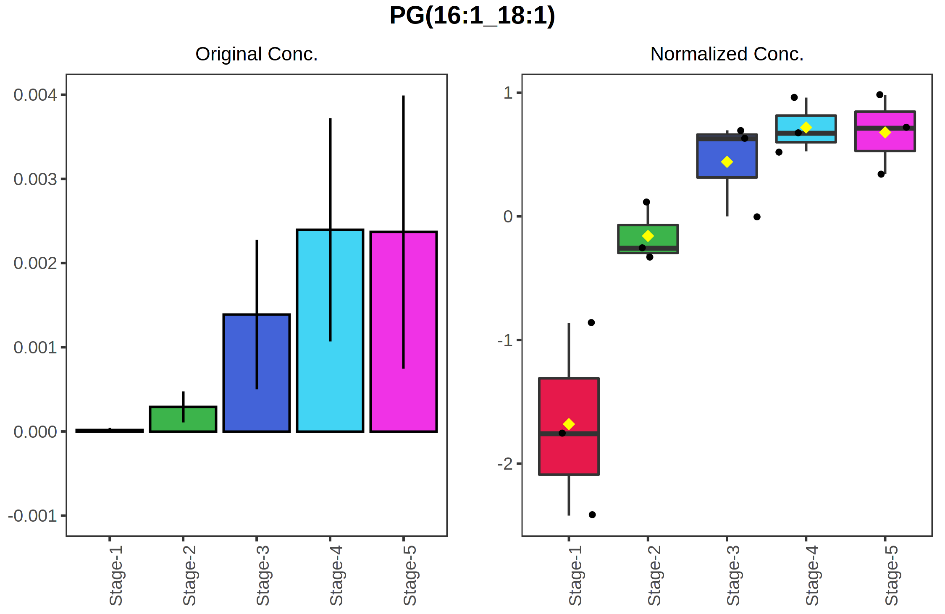

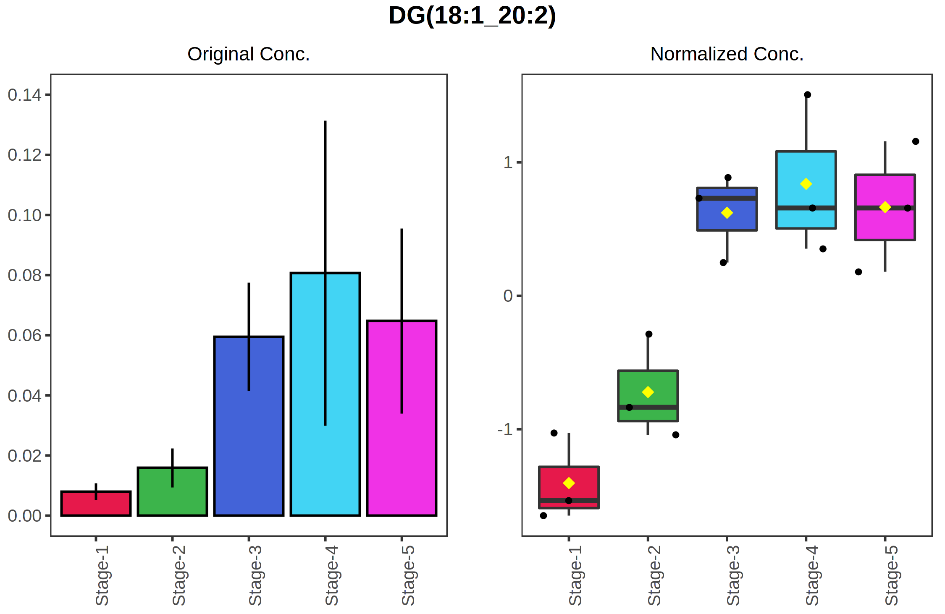

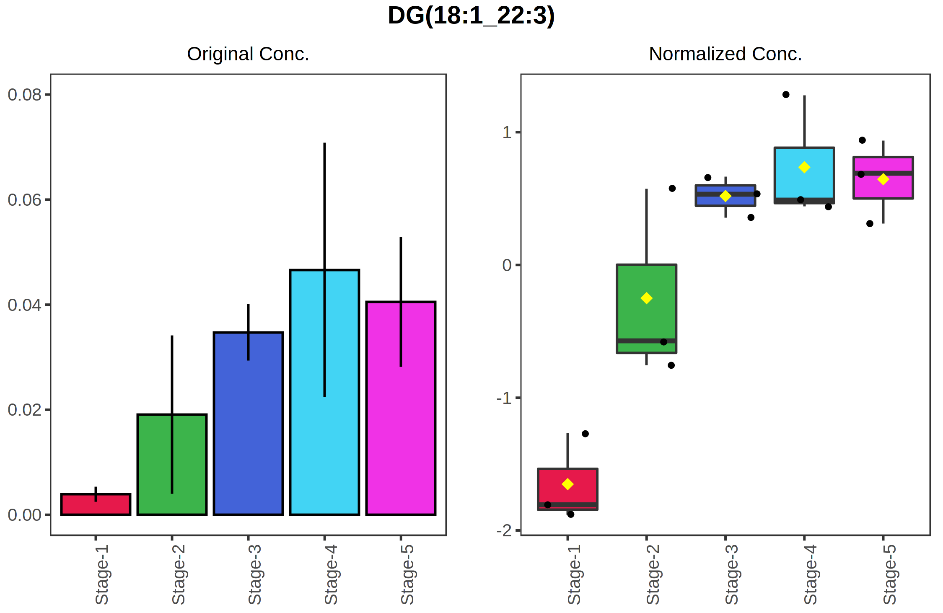

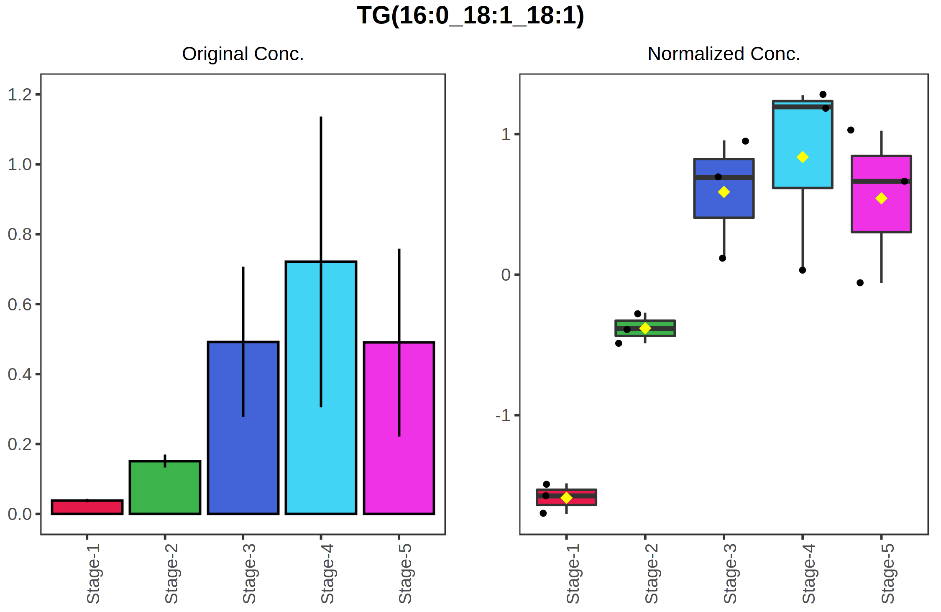

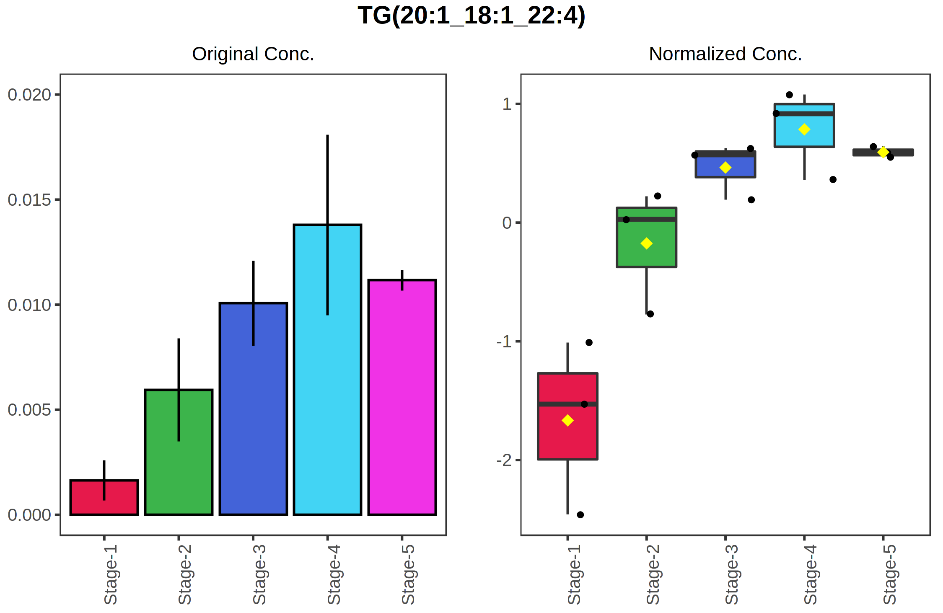

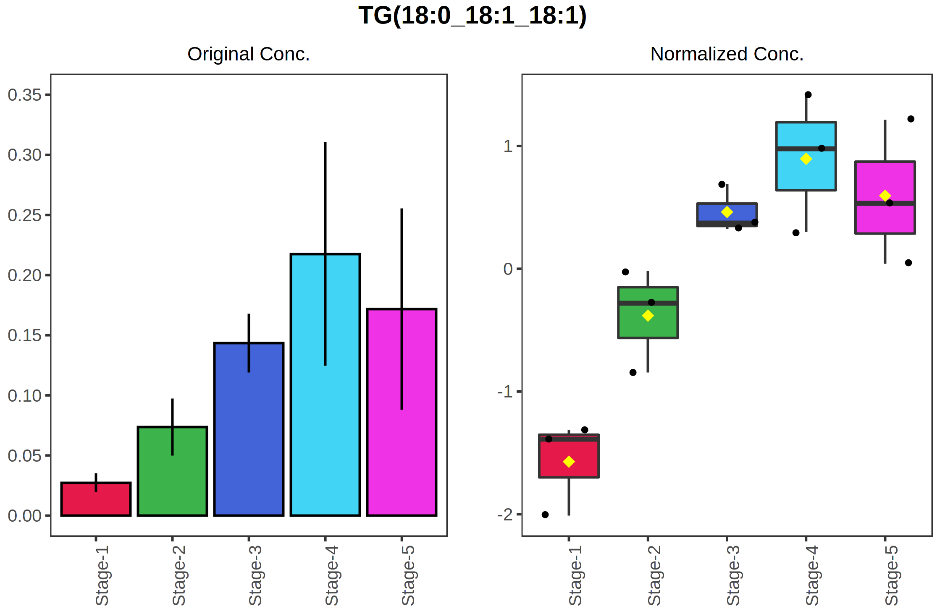


**DG (18:1_20:2)**

**DG (18:1_22:3)**

**TG (16:0_18:1_18:1)**

**TG (20:1_18:1_22:4)**

**TG (18:0_18:1_18:1)**

Normalized concentration

Normalized concentration

Normalized concentration

Normalized concentration

Normalized concentration

**PC (19:0_21:0)**

**PI (16:0_20:3)**

**TG (16:1_18:1_20:3)**

**PG (18:1_18:2)**

**PG (16:1_18:1)**

Normalized concentration

Normalized concentration

Normalized concentration

Normalized concentration

Normalized concentration

**DG (18:1_18:1)**

**TG (16:1_12:1_16:1)**

**TG (19:1_18:1_22:4)**

**LPC (18:1e)**

**DG (20:1_18:1)**

Normalized concentration

Normalized concentration

Normalized concentration

Normalized concentration

Normalized concentration


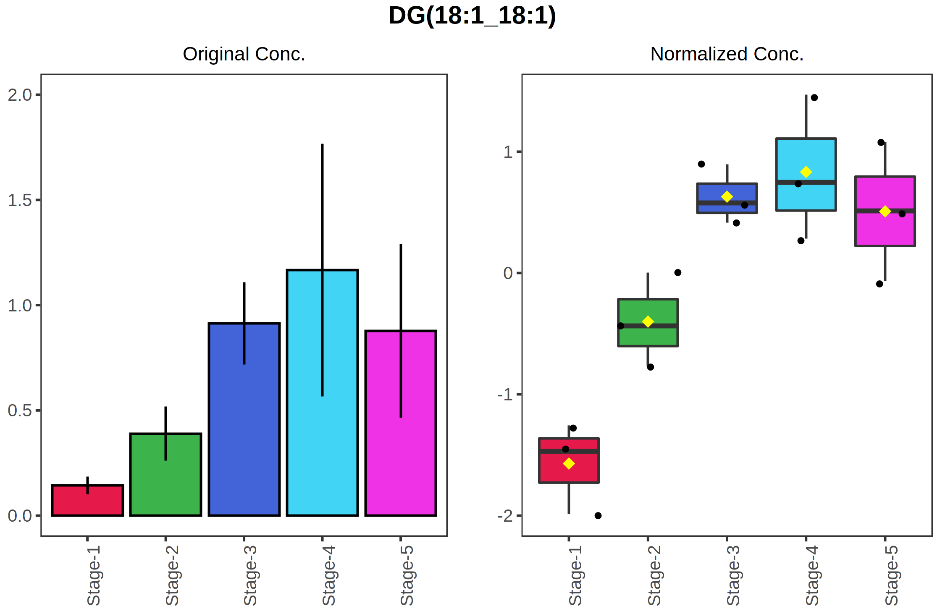

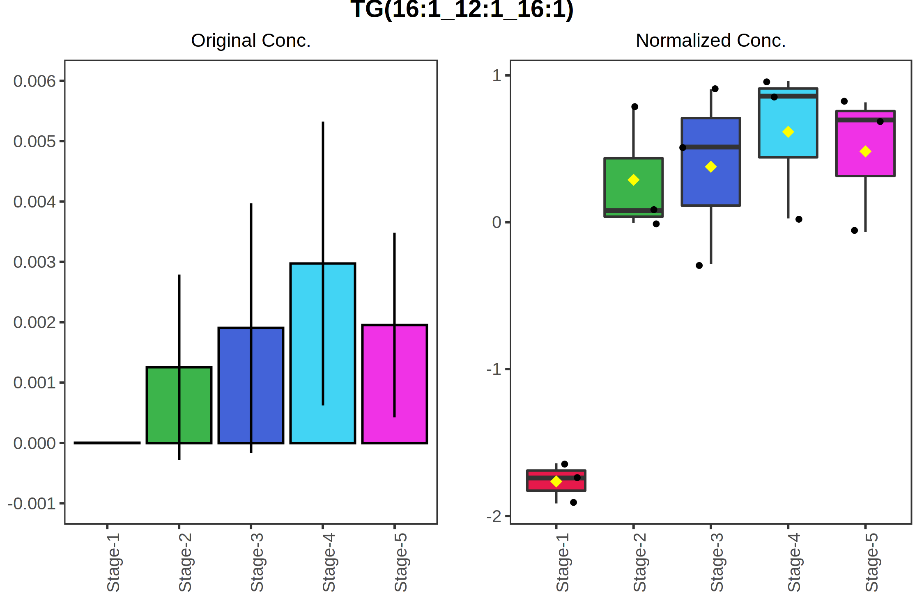

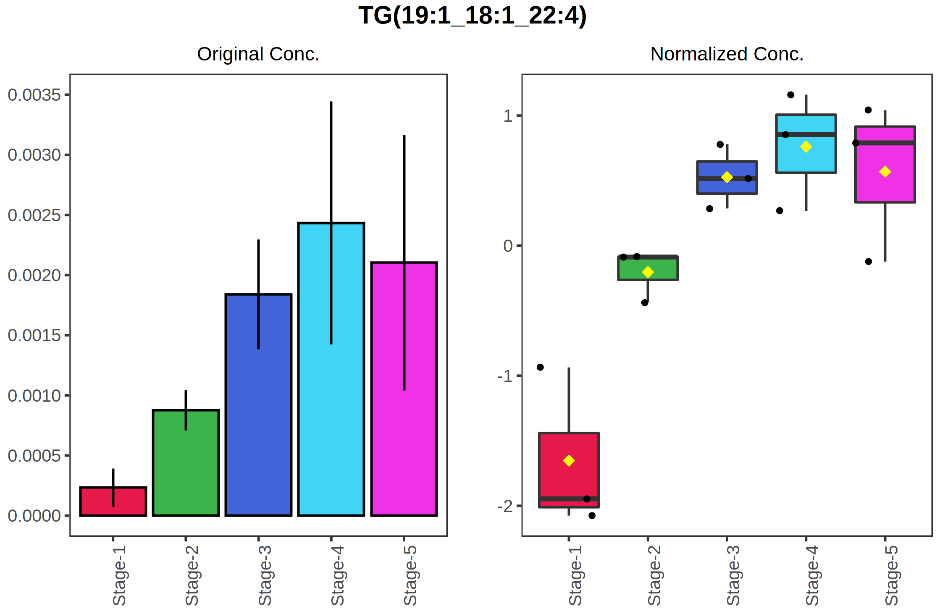

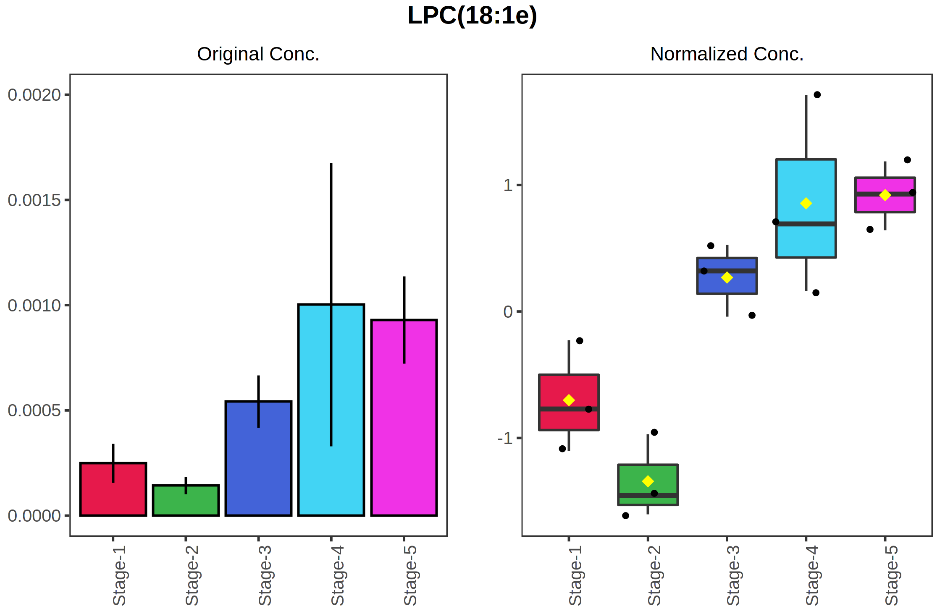

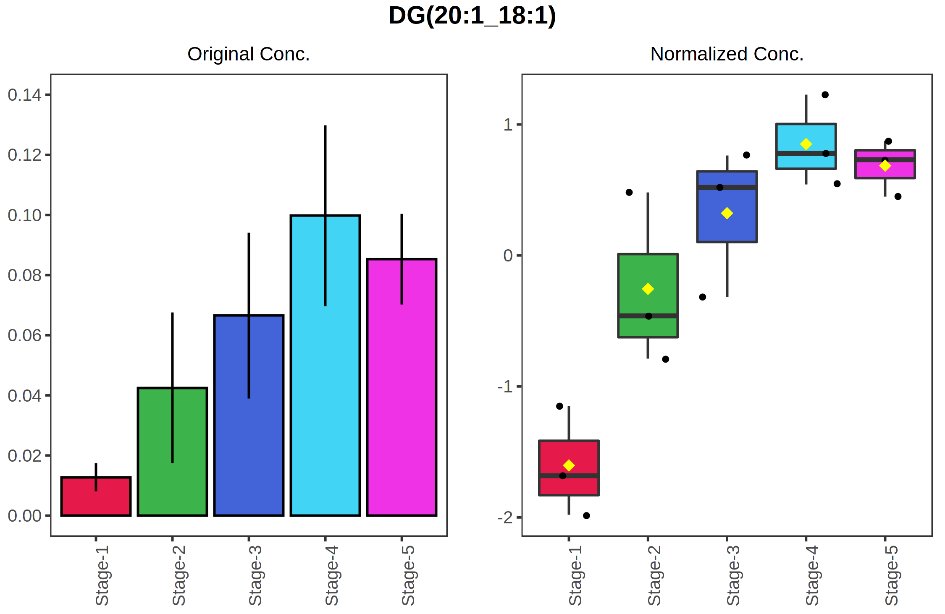

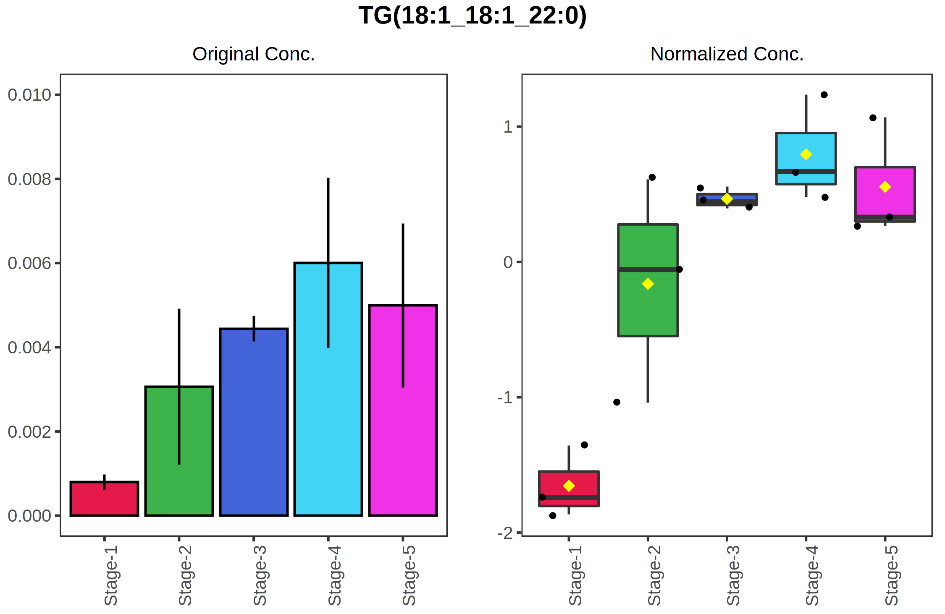

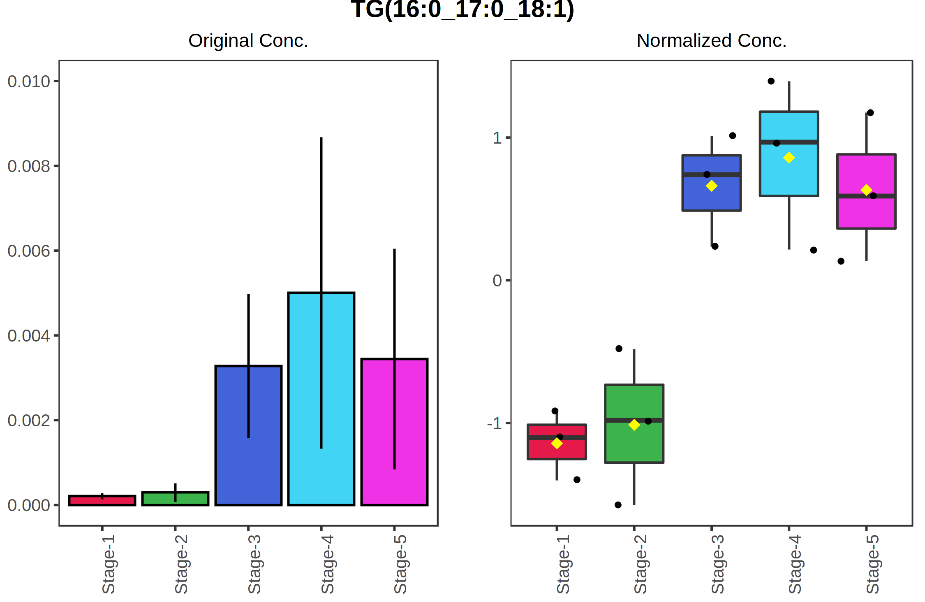

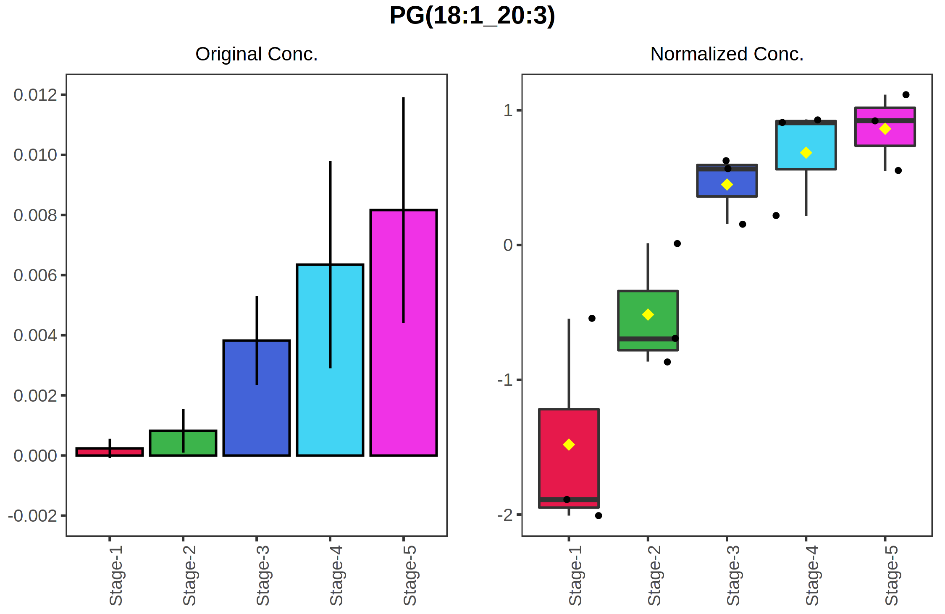

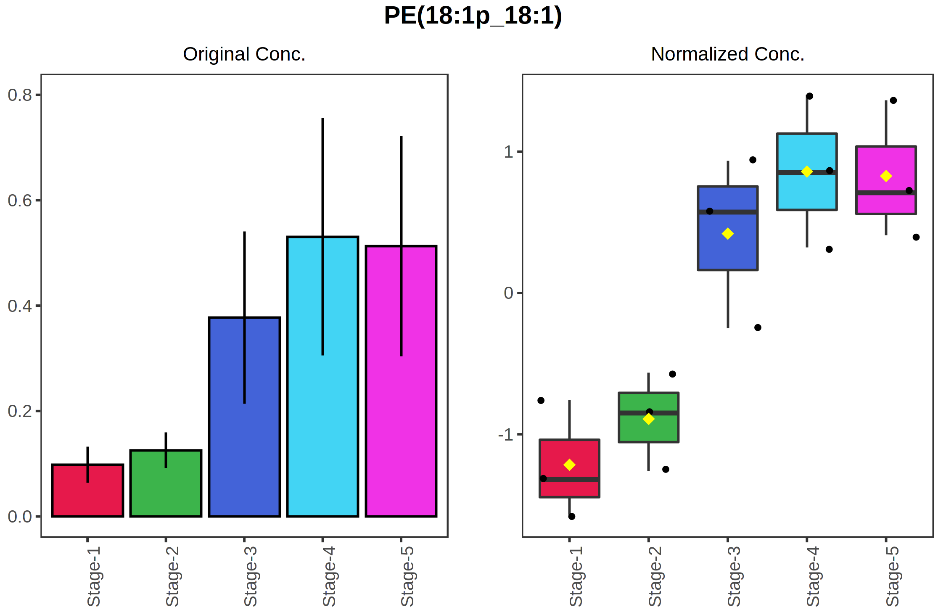

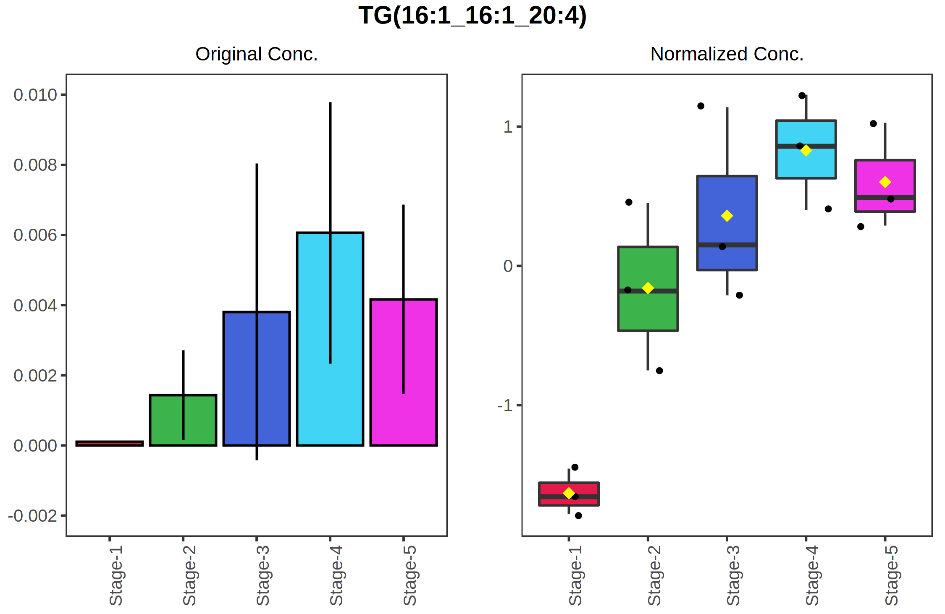


**TG (18:1_18:1_22:0)**

**TG (16:0_17:0_18:1)**

**PG (18:1_20:3)**

**PE (18:1p_18:1)**

**TG (16:1_16:1_20:4)**

Normalized concentration

Normalized concentration

Normalized concentration

Normalized concentration

Normalized concentration
