# Supplementary material for: Data on changes in lipid profiles during the differentiation and maturation of human subcutaneous white adipocytes analyzed using chromatographic and bioinformatic tools
Source: Data Brief. 2022 May 6;42:108245. doi: 10.1016/j.dib.2022.108245 (PMC9114626; doi:10.1016/j.dib.2022.108245)
Supplement: Supplementary file 4 [file mmc4.docx]

**Supplemental Table S2.** Lipid species that were statistically significant.

|  | *f-*value | *P-*value | −log 10 (*P*-value) | FDR | Fisher's LSD |
| --- | --- | --- | --- | --- | --- |
| TG(19:1_18:1_20:1) | 94.771 | 6.58 × 10^-8^ | 7.1815 | 2.03 × 10^-5^ | 2 - 1; 3 - 1; 4 - 1; 5 - 1; 3 - 2; 4 - 2; 5 - 2 |
| TG(18:1_18:1_22:3) | 68.907 | 3.06 × 10^-7^ | 6.5137 | 4.73 × 10^-5^ | 2 - 1; 3 - 1; 4 - 1; 5 - 1; 3 - 2; 4 - 2; 5 - 2 |
| TG(18:1_17:1_20:3) | 57.581 | 7.23 × 10^-7^ | 6.1412 | 7.01 × 10^-5^ | 2 - 1; 3 - 1; 4 - 1; 5 - 1; 3 - 2; 4 - 2; 5 - 2 |
| TG(19:1_18:1_18:1) | 53.951 | 9.84 × 10^-7^ | 6.0068 | 7.01 × 10^-5^ | 2 - 1; 3 - 1; 4 - 1; 5 - 1; 3 - 2; 4 - 2; 5 - 2 |
| TG(19:1_18:1_20:2) | 50.658 | 1.33 × 10^-6^ | 5.8773 | 7.01 × 10^-5^ | 2 - 1; 3 - 1; 4 - 1; 5 - 1; 3 - 2; 4 - 2; 5 - 2 |
| TG(20:1_18:1_18:1) | 50.376 | 1.36 × 10^-6^ | 5.8659 | 7.01 × 10^-5^ | 2 - 1; 3 - 1; 4 - 1; 5 - 1; 3 - 2; 4 - 2; 5 - 2 |
| TG(12:1e_6:0_22:4) | 47.31 | 1.83 × 10^-6^ | 5.7373 | 8.08 × 10^-5^ | 2 - 1; 3 - 1; 4 - 1; 5 - 1; 3 - 2; 4 - 2; 5 - 2; 4 - 3; 5 - 3 |
| TG(18:1_18:1_22:1) | 43.867 | 2.61 × 10^-6^ | 5.5832 | 1.01 × 10^-4^ | 2 - 1; 3 - 1; 4 - 1; 5 - 1; 3 - 2; 4 - 2; 5 - 2 |
| TG(18:1_18:1_18:1) | 37.359 | 5.52 × 10^-6^ | 5.2581 | 1.90 × 10^-4^ | 2 - 1; 3 - 1; 4 - 1; 5 - 1; 3 - 2; 4 - 2; 5 - 2 |
| TG(19:0_18:1_18:1) | 35.057 | 7.41 × 10^-6^ | 5.1304 | 2.14 × 10^-4^ | 2 - 1; 3 - 1; 4 - 1; 5 - 1; 3 - 2; 4 - 2; 5 - 2 |
| TG(18:1_17:1_18:1) | 34.84 | 7.62 × 10^-6^ | 5.118 | 2.14 × 10^-4^ | 2 - 1; 3 - 1; 4 - 1; 5 - 1; 3 - 2; 4 - 2; 5 - 2 |
| TG(18:1_18:1_24:1) | 32.928 | 9.88 × 10^-6^ | 5.0052 | 2.37 × 10^-4^ | 2 - 1; 3 - 1; 4 - 1; 5 - 1 |
| PE(18:1p_22:5) | 32.85 | 9.99 × 10^-6^ | 5.0005 | 2.37 × 10^-4^ | 3 - 1; 4 - 1; 5 - 1; 3 - 2; 4 - 2; 5 - 2 |
| TG(20:1_18:1_22:3) | 28.278 | 1.98 × 10^-5^ | 4.7038 | 4.37 × 10^-4^ | 2 - 1; 3 - 1; 4 - 1; 5 - 1; 4 - 2; 5 - 2 |
| TG(18:1_18:1_22:4) | 27.617 | 2.20 × 10^-5^ | 4.6573 | 4.44 × 10^-4^ | 2 - 1; 3 - 1; 4 - 1; 5 - 1; 3 - 2; 4 - 2; 5 - 2 |
| TG(15:0_18:1_18:1) | 27.166 | 2.37 × 10^-5^ | 4.6251 | 4.44 × 10^-4^ | 2 - 1; 3 - 1; 4 - 1; 5 - 1; 3 - 2; 4 - 2; 5 - 2 |
| TG(18:1_18:1_20:3) | 26.993 | 2.44 × 10^-5^ | 4.6126 | 4.44 × 10^-4^ | 2 - 1; 3 - 1; 4 - 1; 5 - 1; 3 - 2; 4 - 2; 5 - 2 |
| TG(17:0_18:1_18:1) | 26.332 | 2.73 × 10^-5^ | 4.5641 | 4.68 × 10^-4^ | 2 - 1; 3 - 1; 4 - 1; 5 - 1; 3 - 2; 4 - 2; 5 - 2 |
| TG(19:0_18:1_18:3) | 23.279 | 4.73 × 10^-5^ | 4.3251 | 7.67 × 10^-4^ | 2 - 1; 3 - 1; 4 - 1; 5 - 1; 3 - 2; 4 - 2; 5 - 2 |
| TG(16:1_18:1_20:4) | 23.008 | 4.98 × 10^-5^ | 4.3025 | 7.67 × 10^-4^ | 2 - 1; 3 - 1; 4 - 1; 5 - 1; 3 - 2; 4 - 2; 5 - 2 |
| PE(18:1p_20:4) | 22.651 | 5.34 × 10^-5^ | 4.2724 | 7.67 × 10^-4^ | 3 - 1; 4 - 1; 5 - 1; 3 - 2; 4 - 2; 5 - 2; 4 - 3; 5 - 3 |
| TG(17:1_17:1_17:1) | 22.536 | 5.46 × 10^-5^ | 4.2627 | 7.67 × 10^-4^ | 2 - 1; 3 - 1; 4 - 1; 5 - 1; 3 - 2; 4 - 2; 5 - 2 |
| TG(18:1_18:1_18:2) | 21.949 | 6.14 × 10^-5^ | 4.2121 | 7.94 × 10^-4^ | 2 - 1; 3 - 1; 4 - 1; 5 - 1; 3 - 2; 4 - 2; 5 - 2 |
| TG(20:0_18:1_18:1) | 21.922 | 6.17 × 10^-5^ | 4.2098 | 7.94 × 10^-4^ | 2 - 1; 3 - 1; 4 - 1; 5 - 1; 3 - 2; 4 - 2; 5 - 2 |
| DG(18:1_22:4) | 20.895 | 7.62 × 10^-5^ | 4.1183 | 9.41 × 10^-4^ | 2 - 1; 3 - 1; 4 - 1; 5 - 1; 3 - 2; 4 - 2; 5 - 2 |
| PE(18:2e_20:4) | 20.445 | 8.38 × 10^-5^ | 4.077 | 9.91 × 10^-4^ | 3 - 1; 4 - 1; 5 - 1; 3 - 2; 4 - 2; 5 - 2; 4 - 3 |
| ChE(24:6) | 20.184 | 8.86 × 10^-5^ | 4.0526 | 9.91 × 10^-4^ | 1 - 3; 1 - 4; 1 - 5; 2 - 3; 2 - 4; 2 - 5; 3 - 4 |
| TG(16:1_18:1_18:1) | 20.115 | 8.99 × 10^-5^ | 4.0461 | 9.91 × 10^-4^ | 2 - 1; 3 - 1; 4 - 1; 5 - 1; 3 - 2; 4 - 2 |
| TG(18:1_18:1_24:2) | 19.96 | 9.30 × 10^-5^ | 4.0314 | 9.91 × 10^-4^ | 2 - 1; 3 - 1; 4 - 1; 5 - 1 |
| TG(16:0_16:1_17:1) | 19.445 | 1.04 × 10^-4^ | 3.9822 | 1.07 × 10^-3^ | 2 - 1; 3 - 1; 4 - 1; 5 - 1; 3 - 2; 4 - 2; 5 - 2 |
| PC(19:0_21:0) | 18.263 | 1.37 × 10^-4^ | 3.8645 | 1.36 × 10^-3^ | 1 - 3; 1 - 4; 1 - 5; 2 - 3; 2 - 4; 2 - 5 |
| PI(16:0_20:3) | 16.619 | 2.04 × 10^-4^ | 3.6896 | 1.97 × 10^-3^ | 3 - 1; 4 - 1; 5 - 1; 3 - 2; 4 - 2; 5 - 2 |
| TG(16:1_18:1_20:3) | 15.964 | 2.42 × 10^-4^ | 3.6157 | 2.27 × 10^-3^ | 2 - 1; 3 - 1; 4 - 1; 5 - 1; 3 - 2; 4 - 2 |
| PG(18:1_18:2) | 15.63 | 2.65 × 10^-4^ | 3.5771 | 2.31 × 10^-3^ | 2 - 1; 3 - 1; 4 - 1; 5 - 1; 3 - 2; 4 - 2; 5 - 2 |
| PG(16:1_18:1) | 15.569 | 2.69 × 10^-4^ | 3.5701 | 2.31 × 10^-3^ | 2 - 1; 3 - 1; 4 - 1; 5 - 1; 4 - 2; 5 - 2 |
| DG(18:1_20:2) | 15.567 | 2.69 × 10^-4^ | 3.5698 | 2.31 × 10^-3^ | 3 - 1; 4 - 1; 5 - 1; 3 - 2; 4 - 2; 5 - 2 |
| DG(18:1_22:3) | 15.468 | 2.77 × 10^-4^ | 3.5581 | 2.31 × 10^-3^ | 2 - 1; 3 - 1; 4 - 1; 5 - 1; 4 - 2; 5 - 2 |
| TG(16:0_18:1_18:1) | 15.217 | 2.96 × 10^-4^ | 3.5284 | 2.41 × 10^-3^ | 2 - 1; 3 - 1; 4 - 1; 5 - 1; 3 - 2; 4 - 2; 5 - 2 |
| TG(20:1_18:1_22:4) | 14.703 | 3.42 × 10^-4^ | 3.4662 | 2.71 × 10^-3^ | 2 - 1; 3 - 1; 4 - 1; 5 - 1; 4 - 2 |
| TG(18:0_18:1_18:1) | 14.614 | 3.51 × 10^-4^ | 3.4553 | 2.71 × 10^-3^ | 2 - 1; 3 - 1; 4 - 1; 5 - 1; 3 - 2; 4 - 2; 5 - 2 |
| DG(18:1_18:1) | 14.391 | 3.74 × 10^-4^ | 3.4276 | 2.82 × 10^-3^ | 2 - 1; 3 - 1; 4 - 1; 5 - 1; 3 - 2; 4 - 2; 5 - 2 |
| TG(16:1_12:1_16:1) | 13.841 | 4.39 × 10^-4^ | 3.3578 | 3.18 × 10^-3^ | 2 - 1; 3 - 1; 4 - 1; 5 - 1 |
| TG(19:1_18:1_22:4) | 13.811 | 4.43 × 10^-4^ | 3.3539 | 3.18 × 10^-3^ | 2 - 1; 3 - 1; 4 - 1; 5 - 1; 4 - 2 |
| LPC(18:1e) | 13.647 | 4.65 × 10^-4^ | 3.3326 | 3.26 × 10^-3^ | 3 - 1; 4 - 1; 5 - 1; 3 - 2; 4 - 2; 5 - 2 |
| DG(20:1_18:1) | 13.397 | 5.01 × 10^-4^ | 3.2997 | 3.44 × 10^-3^ | 2 - 1; 3 - 1; 4 - 1; 5 - 1; 4 - 2; 5 - 2 |
| TG(18:1_18:1_22:0) | 13.139 | 5.43 × 10^-4^ | 3.2653 | 3.65 × 10^-3^ | 2 - 1; 3 - 1; 4 - 1; 5 - 1; 4 - 2 |
| TG(16:0_17:0_18:1) | 12.851 | 5.94 × 10^-4^ | 3.2262 | 3.91 × 10^-3^ | 3 - 1; 4 - 1; 5 - 1; 3 - 2; 4 - 2; 5 - 2 |
| PG(18:1_20:3) | 12.225 | 7.27 × 10^-4^ | 3.1387 | 4.63 × 10^-3^ | 2 - 1; 3 - 1; 4 - 1; 5 - 1; 3 - 2; 4 - 2; 5 - 2 |
| PE(18:1p_18:1) | 12.174 | 7.39 × 10^-4^ | 3.1314 | 4.63 × 10^-3^ | 3 - 1; 4 - 1; 5 - 1; 3 - 2; 4 - 2; 5 - 2 |
| TG(16:1_16:1_20:4) | 12.133 | 7.49 × 10^-4^ | 3.1255 | 4.63 × 10^-3^ | 2 - 1; 3 - 1; 4 - 1; 5 - 1; 4 - 2 |
| TG(16:0_16:1_17:0) | 11.905 | 8.08 × 10^-4^ | 3.0925 | 4.90 × 10^-3^ | 3 - 1; 4 - 1; 5 - 1; 3 - 2; 4 - 2; 5 - 2 |
| PI(18:1_18:1) | 11.706 | 8.64 × 10^-4^ | 3.0634 | 5.14 × 10^-3^ | 3 - 1; 4 - 1; 5 - 1; 3 - 2; 4 - 2; 5 - 2 |
| TG(18:1_18:1_22:2) | 11.432 | 9.49 × 10^-4^ | 3.0226 | 5.53 × 10^-3^ | 2 - 1; 3 - 1; 4 - 1; 5 - 1 |
| PI(16:0_20:4) | 11.329 | 9.84 × 10^-4^ | 3.0069 | 5.63 × 10^-3^ | 3 - 1; 4 - 1; 5 - 1; 3 - 2; 4 - 2; 5 - 2 |
| TG(16:1_16:1_18:1) | 11.24 | 1.02 × 10^-3^ | 2.9935 | 5.69 × 10^-3^ | 2 - 1; 3 - 1; 4 - 1; 5 - 1 |
| TG(16:0_16:1_18:1) | 11.197 | 1.03 × 10^-3^ | 2.987 | 5.69 × 10^-3^ | 2 - 1; 3 - 1; 4 - 1; 5 - 1; 4 - 2 |
| PI(16:0_18:1) | 11.107 | 1.06 × 10^-3^ | 2.9731 | 5.77 × 10^-3^ | 3 - 1; 4 - 1; 5 - 1; 3 - 2; 4 - 2; 5 - 2 |
| TG(19:1_18:1_20:3) | 10.853 | 1.16 × 10^-3^ | 2.9337 | 6.21 × 10^-3^ | 2 - 1; 3 - 1; 4 - 1; 5 - 1 |
| DG(18:1_18:2) | 10.802 | 1.19 × 10^-3^ | 2.9258 | 6.21 × 10^-3^ | 3 - 1; 4 - 1; 5 - 1; 3 - 2; 4 - 2; 5 - 2 |
| PE(16:1e_20:4) | 10.659 | 1.25 × 10^-3^ | 2.903 | 6.44 × 10^-3^ | 3 - 1; 4 - 1; 5 - 1; 3 - 2; 4 - 2; 5 - 2 |
| TG(16:1_14:1_16:1) | 10.415 | 1.37 × 10^-3^ | 2.864 | 6.93 × 10^-3^ | 2 - 1; 3 - 1; 4 - 1; 5 - 1 |
| TG(16:1_14:1_18:2) | 10.151 | 1.51 × 10^-3^ | 2.8209 | 7.33 × 10^-3^ | 2 - 1; 3 - 1; 4 - 1; 5 - 1 |
| DG(16:1_14:1) | 10.144 | 1.51 × 10^-3^ | 2.8198 | 7.33 × 10^-3^ | 3 - 1; 4 - 1; 5 - 1; 3 - 2; 4 - 2; 5 - 2 |
| TG(16:1_16:1_17:1) | 10.139 | 1.52 × 10^-3^ | 2.8189 | 7.33 × 10^-3^ | 2 - 1; 3 - 1; 4 - 1; 5 - 1; 4 - 2 |
| PC(18:3e_18:1) | 10.042 | 1.57 × 10^-3^ | 2.8028 | 7.49 × 10^-3^ | 3 - 1; 4 - 1; 5 - 1; 4 - 2; 5 - 2 |
| PE(14:0p_22:4) | 9.8708 | 1.68 × 10^-3^ | 2.7742 | 7.87 × 10^-3^ | 3 - 1; 4 - 1; 5 - 1; 3 - 2; 4 - 2; 5 - 2 |
| TG(16:1_16:1_18:2) | 9.7016 | 1.80 × 10^-3^ | 2.7455 | 8.18 × 10^-3^ | 2 - 1; 3 - 1; 4 - 1; 5 - 1 |
| PG(18:0_18:1) | 9.6968 | 1.80 × 10^-3^ | 2.7447 | 8.18 × 10^-3^ | 3 - 1; 4 - 1; 5 - 1; 3 - 2; 4 - 2; 5 - 2 |
| TG(16:1_16:1_18:3) | 9.6328 | 1.85 × 10^-3^ | 2.7338 | 8.27 × 10^-3^ | 2 - 1; 3 - 1; 4 - 1; 5 - 1; 4 - 2 |
| PE(18:2e_22:4) | 9.5371 | 1.92 × 10^-3^ | 2.7173 | 8.46 × 10^-3^ | 3 - 1; 4 - 1; 5 - 1; 3 - 2; 4 - 2; 5 - 2 |
| TG(16:1_18:1_18:2) | 9.4316 | 2.00 × 10^-3^ | 2.699 | 8.54 × 10^-3^ | 2 - 1; 3 - 1; 4 - 1; 5 - 1 |
| TG(16:1_16:1_20:3) | 9.4292 | 2.00 × 10^-3^ | 2.6986 | 8.54 × 10^-3^ | 2 - 1; 3 - 1; 4 - 1; 5 - 1 |
| PG(18:1_18:1) | 9.4088 | 2.02 × 10^-3^ | 2.695 | 8.54 × 10^-3^ | 2 - 1; 3 - 1; 4 - 1; 5 - 1; 4 - 2; 5 - 2 |
| TG(18:1_22:1_22:5) | 9.1908 | 2.20 × 10^-3^ | 2.6566 | 9.21 × 10^-3^ | 2 - 1; 3 - 1; 4 - 1; 5 - 1 |
| PI(18:1_20:4) | 9.0832 | 2.30 × 10^-3^ | 2.6374 | 9.29 × 10^-3^ | 3 - 1; 4 - 1; 5 - 1; 3 - 2; 4 - 2; 5 - 2 |
| PE(18:1_22:3) | 9.0766 | 2.31 × 10^-3^ | 2.6362 | 9.29 × 10^-3^ | 1 - 2; 1 - 3; 2 - 3; 5 - 2; 4 - 3; 5 - 3 |
| TG(16:0_16:1_16:1) | 9.0543 | 2.33 × 10^-3^ | 2.6322 | 9.29 × 10^-3^ | 2 - 1; 3 - 1; 4 - 1; 5 - 1 |
| PE(18:1p_16:1) | 9.0399 | 2.35 × 10^-3^ | 2.6296 | 9.29 × 10^-3^ | 3 - 1; 4 - 1; 5 - 1; 3 - 2; 4 - 2; 5 - 2 |
| PS(20:1_18:1) | 8.9865 | 2.40 × 10^-3^ | 2.62 | 9.38 × 10^-3^ | 2 - 1; 3 - 1; 4 - 1; 5 - 1; 3 - 2; 4 - 2 |
| PG(18:1_20:2) | 8.8967 | 2.49 × 10^-3^ | 2.6037 | 9.62 × 10^-3^ | 2 - 1; 3 - 1; 4 - 1; 5 - 1 |
| PE(18:1p_20:1) | 8.6843 | 2.72 × 10^-3^ | 2.5647 | 1.04 × 10^-2^ | 3 - 1; 4 - 1; 5 - 1; 3 - 2; 4 - 2; 5 - 2 |
| LPC(16:1e) | 8.5049 | 2.94 × 10^-3^ | 2.5312 | 1.01 × 10^-2^ | 4 - 1; 5 - 1; 3 - 2; 4 - 2; 5 - 2; 5 - 3 |
| LPE(16:1e) | 8.5018 | 2.95 × 10^-3^ | 2.5306 | 1.01 × 10^-2^ | 3 - 1; 4 - 1; 5 - 1; 3 - 2; 4 - 2; 5 - 2 |
| TG(16:0_16:0_16:1) | 8.4245 | 3.05 × 10^-3^ | 2.516 | 1.12 × 10^-2^ | 3 - 1; 4 - 1; 5 - 1; 4 - 2; 5 - 2 |
| TG(26:1_18:1_18:1) | 8.2633 | 3.27 × 10^-3^ | 2.4853 | 1.19 × 10^-2^ | 2 - 1; 3 - 1; 4 - 1; 5 - 1 |
| TG(16:1_16:1_22:5) | 8.1018 | 3.52 × 10^-3^ | 2.4541 | 1.24 × 10^-2^ | 3 - 1; 4 - 1; 5 - 1; 4 - 2; 5 - 2 |
| TG(18:1_14:0_14:0) | 8.0968 | 3.52 × 10^-3^ | 2.4531 | 1.24 × 10^-2^ | 2 - 1; 3 - 1; 4 - 1; 5 - 1; 4 - 2 |
| ChE(20:4) | 8.079 | 3.55× 10^-3^ | 2.4496 | 1.24 × 10^-2^ | 1 - 2; 3 - 2; 4 - 2; 5 - 2 |
| PE(18:2e_22:6) | 8.0722 | 3.56 × 10^-3^ | 2.4483 | 1.24 × 10^-2^ | 3 - 1; 4 - 1; 5 - 1; 3 - 2; 4 - 2; 5 - 2 |
| TG(16:0_16:0_18:1) | 7.9641 | 3.74 × 10^-3^ | 2.4271 | 1.28 × 10^-2^ | 3 - 1; 4 - 1; 5 - 1; 3 - 2; 4 - 2; 5 - 2 |
| LPC(18:0) | 7.865 | 3.91 × 10^-3^ | 2.4074 | 1.33 × 10^-2^ | 1 - 2; 5 - 1; 4 - 2; 5 - 2; 4 - 3; 5 - 3 |
| PE(18:1_22:1) | 7.5899 | 4.45 × 10^-3^ | 2.352 | 1.49 × 10^-2^ | 2 - 1; 3 - 1; 4 - 1; 5 - 1; 4 - 2 |
| PE(18:0p_22:5) | 7.5673 | 4.49 × 10^-3^ | 2.3474 | 1.49 × 10^-2^ | 4 - 1; 5 - 1; 3 - 2; 4 - 2; 5 - 2 |
| PC(12:0e_6:0) | 7.3823 | 4.91 × 10^-3^ | 2.3093 | 1.60 × 10^-2^ | 1 - 2; 3 - 2; 4 - 2; 5 - 2 |
| TG(15:0_15:0_15:0) | 7.375 | 4.92 × 10^-3^ | 2.3078 | 1.60 × 10^-2^ | 3 - 1; 4 - 1; 5 - 1; 4 - 2; 5 - 2; 4 - 3 |
| DG(18:1_20:3) | 7.2307 | 5.28 × 10^-3^ | 2.2775 | 1.70 × 10^-2^ | 3 - 1; 4 - 1; 5 - 1; 3 - 2; 4 - 2; 5 - 2 |
| PC(22:2_22:3) | 7.0721 | 5.70 × 10^-3^ | 2.2438 | 1.82 × 10^-2^ | 1 - 3; 1 - 4; 1 - 5; 2 - 4; 2 - 5 |
| PE(18:1_22:5) | 7.0192 | 5.86 × 10^-3^ | 2.2324 | 1.85 × 10^-2^ | 2 - 1; 3 - 1; 4 - 1; 5 - 1; 4 - 5 |
| TG(16:1_9:0_18:1) | 6.9162 | 6.16 × 10^-3^ | 2.2101 | 1.92 × 10^-2^ | 2 - 1; 3 - 1; 4 - 1; 5 - 1 |
| TG(16:0_14:1_16:1) | 6.7831 | 6.59 × 10^-3^ | 2.181 | 2.02 × 10^-2^ | 2 - 1; 3 - 1; 4 - 1; 5 - 1 |
| TG(16:1_14:0_14:1) | 6.7794 | 6.60 × 10^-3^ | 2.1802 | 2.02 × 10^-2^ | 2 - 1; 3 - 1; 4 - 1; 5 - 1 |
| TG(16:1_18:1_22:6) | 6.6698 | 6.98 × 10^-3^ | 2.1559 | 2.12 × 10^-2^ | 2 - 1; 3 - 1; 4 - 1; 5 - 1; 4 - 2 |
| TG(18:1_18:1_20:2) | 6.6255 | 7.15 × 10^-3^ | 2.146 | 2.14 × 10^-2^ | 3 - 1; 4 - 1; 5 - 1; 3 - 2; 4 - 2; 5 - 2 |
| DG(16:1_18:1) | 6.5526 | 7.42 × 10^-3^ | 2.1296 | 2.18 × 10^-2^ | 2 - 1; 3 - 1; 4 - 1; 5 - 1 |
| TG(16:0_14:0_16:0) | 6.5518 | 7.42 × 10^-3^ | 2.1294 | 2.18 × 10^-2^ | 3 - 1; 4 - 1; 5 - 1; 4 - 2; 5 - 2 |
| PE(18:0_22:6) | 6.5341 | 7.49 × 10^-3^ | 2.1254 | 2.18 × 10^-2^ | 1 - 3; 1 - 4; 1 - 5; 2 - 5 |
| PI(16:1_18:1) | 6.5046 | 7.61 × 10^-3^ | 2.1188 | 2.18 × 10^-2^ | 3 - 1; 4 - 1; 5 - 1; 3 - 2; 4 - 2 |
| TG(16:1_16:1_16:1) | 6.5011 | 7.62 × 10^-3^ | 2.1179 | 2.18 × 10^-2^ | 2 - 1; 3 - 1; 4 - 1; 5 - 1 |
| PE(20:1_18:1) | 6.4221 | 7.94 × 10^-3^ | 2.1 | 2.25 × 10^-2^ | 3 - 1; 4 - 1; 5 - 1; 4 - 2 |
| PE(18:0p_22:3) | 6.3594 | 8.21 × 10^-3^ | 2.0856 | 2.30 × 10^-2^ | 3 - 1; 4 - 1; 5 - 1 |
| TG(16:1_16:1_22:6) | 6.3506 | 8.25 × 10^-3^ | 2.0836 | 2.30 × 10^-2^ | 3 - 1; 4 - 1; 5 - 1; 4 - 2 |
| PC(19:1_23:1) | 6.2755 | 8.59 × 10^-3^ | 2.0662 | 2.37 × 10^-2^ | 1 - 4; 1 - 5; 2 - 3; 2 - 4; 2 - 5 |
| DG(15:0_15:0) | 6.2012 | 8.93 × 10^-3^ | 2.0489 | 2.44 × 10^-2^ | 3 - 1; 4 - 1; 5 - 1; 3 - 2; 4 - 2; 5 - 2 |
| PC(17:1_18:1) | 6.1523 | 9.17 × 10^-3^ | 2.0374 | 2.49 × 10^-2^ | 3 - 1; 4 - 1; 5 - 1; 4 - 2 |
| PE(16:1e_18:1) | 6.0484 | 9.17 × 10^-3^ | 2.0129 | 2.61 × 10^-2^ | 3 - 1; 4 - 1; 5 - 1; 3 - 2; 4 - 2; 5 - 2 |
| LPC(17:0) | 6.0326 | 9.79 × 10^-3^ | 2.0091 | 2.61 × 10^-2^ | 1 - 2; 4 - 2; 5 - 2; 5 - 3 |
| PE(18:1p_22:6) | 5.9703 | 1.01 × 10^-2^ | 1.9942 | 2.67 × 10^-2^ | 3 - 1; 4 - 1; 5 - 1; 3 - 2; 4 - 2 |
| TG(14:0_14:0_14:0) | 5.958 | 1.02 × 10^-2^ | 1.9913 | 2.67 × 10^-2^ | 3 - 1; 4 - 1; 5 - 1; 4 - 2; 5 - 2 |
| TG(16:1_12:0_14:0) | 5.9444 | 1.03 × 10^-2^ | 1.988 | 2.67 × 10^-2^ | 3 - 1; 4 - 1; 5 - 1; 4 - 2 |
| TG(16:1_14:0_14:0) | 5.9263 | 1.04 × 10^-2^ | 1.9837 | 2.67 × 10^-2^ | 3 - 1; 4 - 1; 5 - 1; 4 - 2 |
| DG(16:1_18:2) | 5.9058 | 1.05 × 10^-2^ | 1.9787 | 2.68 × 10^-2^ | 3 - 1; 4 - 1; 5 - 1; 3 - 2; 4 - 2 |
| TG(15:0_16:1_16:1) | 5.8567 | 1.08 × 10^-2^ | 1.9669 | 2.71 × 10^-2^ | 3 - 1; 4 - 1; 5 - 1; 4 - 2; 5 - 2 |
| TG(16:0_6:0_18:1) | 5.8448 | 1.09 × 10^-2^ | 1.964 | 2.71 × 10^-2^ | 3 - 1; 4 - 1; 5 - 1; 4 - 2; 5 - 2 |
| PE(18:0_22:5) | 5.8437 | 1.09 × 10^-2^ | 1.9637 | 2.71 × 10^-2^ | 4 - 1; 4 - 2; 5 - 2; 4 - 3; 5 - 3 |
| TG(16:0_9:0_16:1) | 5.3527 | 1.44 × 10^-2^ | 1.8411 | 3.55 × 10^-2^ | 3 - 1; 4 - 1; 5 - 1 |
| TG(16:0_14:0_14:0) | 5.3468 | 1.45 × 10^-2^ | 1.8396 | 3.55 × 10^-2^ | 3 - 1; 4 - 1; 5 - 1; 4 - 2; 5 - 2 |
| LPE(15:0) | 5.3168 | 1.47 × 10^-2^ | 1.8319 | 3.58 × 10^-2^ | 3 - 1; 4 - 1; 5 - 1; 3 - 2; 4 - 2; 5 - 2 |
| PE(18:1e_18:1) | 5.2097 | 1.57 × 10^-2^ | 1.8041 | 3.77 × 10^-2^ | 3 - 1; 4 - 1; 5 - 1; 4 - 2; 5 - 2 |
| PE(18:1p_16:0) | 5.2033 | 1.58 × 10^-2^ | 1.8025 | 3.77 × 10^-2^ | 3 - 1; 4 - 1; 5 - 1; 4 - 2; 5 - 2 |
| PC(16:1e_16:0) | 5.1676 | 1.61 × 10^-2^ | 1.7931 | 3.83 × 10^-2^ | 1 - 2; 1 - 3; 4 - 2; 5 - 2 |
| PS(18:0_16:0) | 5.1484 | 1.63 × 10^-2^ | 1.7881 | 3.84 × 10^-2^ | 1 - 3; 1 - 4; 1 - 5 |
| LPC(19:1) | 5.0915 | 1.69 × 10^-2^ | 1.7731 | 3.95 × 10^-2^ | 4 - 1; 5 - 1; 4 - 2; 5 - 2 |
| DG(16:0_18:1) | 5.0775 | 1.70 × 10^-2^ | 1.7694 | 3.95 × 10^-2^ | 3 - 1; 4 - 1; 5 - 1; 3 - 2; 4 - 2; 5 - 2 |
| PE(18:2e_22:3) | 5.0388 | 1.74 × 10^-2^ | 1.7591 | 4.01 × 10^-2^ | 3 - 1; 4 - 1; 5 - 1; 3 - 2; 4 - 2; 5 - 2 |
| PE(16:0p_20:3) | 5.0305 | 1.75 × 10^-2^ | 1.7569 | 4.01 × 10^-2^ | 3 - 1; 4 - 1; 5 - 1; 4 - 2; 5 - 2 |
| ChE(18:1) | 5.0107 | 1.77 × 10^-2^ | 1.7517 | 4.03 × 10^-2^ | 1 - 2; 3 - 2; 4 - 2; 5 - 2 |
| TG(18:1_18:1_22:5) | 4.9107 | 1.88 × 10^-2^ | 1.7248 | 4.25 × 10^-2^ | 3 - 1; 4 - 1; 5 - 1 |
| TG(18:1_22:4_22:4) | 4.8635 | 1.94 × 10^-2^ | 1.712 | 4.35 × 10^-2^ | 3 - 1; 4 - 1; 5 - 1 |
| TG(16:0_10:1_16:1) | 4.8438 | 1.96 × 10^-2^ | 1.7067 | 4.35 × 10^-2^ | 3 - 1; 4 - 1; 5 - 1 |
| PG(18:1_22:4) | 4.8388 | 1.97 × 10^-2^ | 1.7053 | 4.35 × 10^-2^ | 3 - 1; 4 - 1; 5 - 1; 4 - 2 |
| TG(12:0e_6:0_20:4) | 4.8266 | 1.99 × 10^-2^ | 1.702 | 4.35 × 10^-2^ | 1 - 2; 4 - 2; 5 - 2 |
| DG(16:1_16:1) | 4.7229 | 2.12 × 10^-2^ | 1.6735 | 4.61 × 10^-2^ | 3 - 1; 4 - 1; 5 - 1 |
| PE(16:0p_20:1) | 4.7035 | 2.15 × 10^-2^ | 1.6681 | 4.64 × 10^-2^ | 3 - 1; 4 - 1; 5 - 1; 4 - 2; 5 - 2 |
| LPC(14:0) | 4.6496 | 2.22 × 10^-2^ | 1.6531 | 4.77 × 10^-2^ | 3 - 1; 4 - 1; 5 - 1; 5 - 2 |
| DG(18:1_20:4) | 4.6027 | 2.29 × 10^-2^ | 1.64 | 4.88 × 10^-2^ | 4 - 1; 5 - 1; 4 - 2; 5 - 2 |

Data were analyzed using the one-way ANOVA followed by Fisher's least significant difference (LSD) post hoc tests (FDR < 0.05). The numbers in the Fisher's LSD column indicate the stage.
